# A New Benzofuran Glycoside and Indole Alkaloids from a Sponge-Associated Rare Actinomycete, *Amycolatopsis* sp.

**DOI:** 10.3390/md12042326

**Published:** 2014-04-22

**Authors:** Yun Kwon, Seong-Hwan Kim, Yoonho Shin, Munhyung Bae, Byung-Yong Kim, Sang Kook Lee, Ki-Bong Oh, Jongheon Shin, Dong-Chan Oh

**Affiliations:** 1 Natural Products Research Institute, College of Pharmacy, Seoul National University, Seoul 151-742, Korea; E-Mails: kisi2016@snu.ac.kr (Y.K.); yanberk@snu.ac.kr (S.-H.K.); dicafree5@snu.ac.kr (Y.S.); baemoon89@snu.ac.kr (M.B.); sklee61@snu.ac.kr (S.K.L.); shinj@snu.ac.kr (J.S.); 2 ChunLab, Inc., Seoul National University, Seoul, 151-742, Korea; E-Mail: greg6044@gmail.com; 3 Department of Agricultural Biotechnology, College of Agriculture and Life Science, Seoul National University, Seoul 151-921, Korea; E-Mail: ohkibong@snu.ac.kr

**Keywords:** sponge, rare actinomycete, *Amycolatopsis*, benzofuran, cyclopiazonic acid

## Abstract

Three new secondary metabolites, amycofuran (**1**), amycocyclopiazonic acid (**2**), and amycolactam (**3**), were isolated from the sponge-associated rare actinomycete *Amycolatopsis* sp. Based on combined spectroscopic analyses, the structures of **1**–**3** were determined to be a new benzofuran glycoside and new indole alkaloids related to cyclopiazonic acids, a class that has previously only been reported in fungi. The absolute configurations of **1** and **3** were deduced by ECD calculations, whereas that of **2** was determined using the modified Mosher method. Amycolactam (**3**) displayed significant cytotoxicity against the gastric cancer cell line SNU638 and the colon cancer cell line HCT116.

## 1. Introduction

Natural products from marine organisms have been proposed as “a new wave of drugs” because of their great potential for supplying structurally and biologically novel compounds for drug discovery [1]. As of 2013, several drugs derived from marine natural products are in clinical use. These drugs include the anticancer drug cytarabine and the antiviral agent vidarabine from the sponge *Tethya crypta*; Prialt from the cone snail *Conus magnus*, which is used for chronic pain treatment; the anticancer drug Yondelis from the marine tunicate *Ecteinascidia turbinata*; and Halaven from the sponge *Halichondria okadai*, a compound used to treat breast cancer [2]. During drug development, compound supply was one of the most challenging problems to overcome, as these drugs originate from marine invertebrates, which are difficult to cultivate [1]. The recent advent of the genomic era and the development of microbial biotechnology have revealed that the majority of secondary metabolites from marine invertebrates are actually produced by symbiotic microorganisms in invertebrate tissues, indicating that the microbial fermentation of useful marine invertebrate-derived compounds may resolve the supply problem [3]. For example, a recent study determined that didemnin anticancer agents, originally discovered from the tunicate *Trididemnum solidum*, were produced by the symbiotic bacteria *Tistrella mobilis* and *Tistrella bauzanensis* [4]. The sponge-derived metabolites swinholide A [5,6], onnamide A [7], and psymberin [8] were also shown to be bacterially produced natural products. These results have spurred the study of the chemistry and phylogenetic diversity of symbiotic microbial communities in marine invertebrates [9,10].

**Figure 1:**
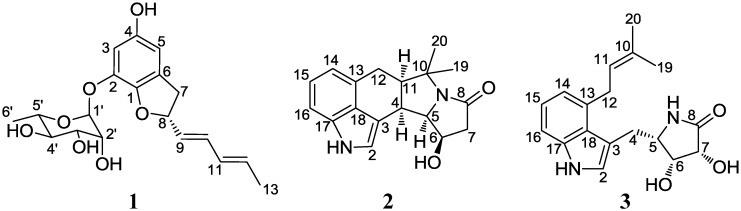
The structures of amycofuran (**1**), amycocyclopiazonic acid (**2**), and amycolactam (**3**).

Actinomycetes, belonging to the phylum Actinobacteria, are prolific chemical synthesizers, providing 40% of the 33,500 microbial bioactive compounds reported as of 2010, including numerous drugs in clinical use [11]. Actinomycetes are one of the major phylogenetic groups in symbiosis with sponges by accounting for ~15% of all symbiotic communities associated with sponges [9]. Given that actinomycetes are chemically prolific and that sponge-associated actinomycetes have not been thoroughly studied, chemical investigations of sponge-associated actinomycetes should lead to the discovery of a novel chemical hemisphere [12]. However, compared to the phylogenetic analysis of sponge-associated microbial communities, the chemical investigation of sponge-associated actinomycetes is only just beginning, as demonstrated by a few recent examples, including new nucleoside analogs discovered from *Streptomyces microflavus* in association with the sponge *Hymeniacidon perlevis* [13] and new lobophorin derivatives isolated from a sponge-associated actinomycete, *Streptomyces carnosus* [14]. Therefore, we selectively isolated actinomycete strains associated with marine sponges to search for new bioactive compounds. Then, we chemically analyzed the production of secondary metabolites from sponge-associated actinomycetes by LC/MS (Liquid Chromatography/Mass Spectrometry). During our chemical screening, we found that a rare actinomycete strain (Cra33g) belonging to *Amycolatopsis* sp., produced a new benzofuran glycoside, as well as two new indole alkaloids, which are quite rare as bacterial metabolites. Here, we report the structural elucidation and biological activity of these three new compounds from the *Amycolatopsis* strain: amycofuran (**1**), amycocyclopiazonic acid (**2**), and amycolactam (**3**) (Figure 1).

## 2. Results and Discussion

### 2.1. Structural Elucidation

**Figure 2:**
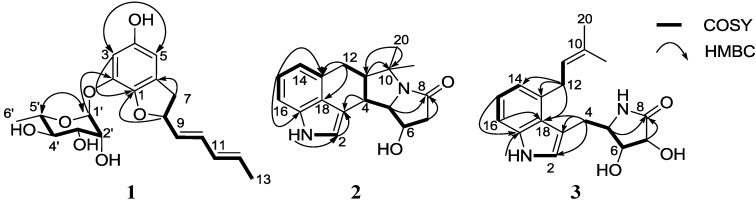
Key ^1^H–^1^H COSY and HMBC correlations of **1**–**3**.

Amycofuran (**1**) was isolated as a white powder, and the molecular formula was established as C_19_H_24_O_7_ by HR-ESI (High Resolution-Electrospray Ionization) mass (obsd [M + Na]^+^ *m*/*z* 387.1407; calcd [M + Na]^+^ 387.1420) and ^1^H and ^13^C NMR spectroscopic data (Table 1). The ^1^H and HSQC (Heteronuclear Single Quantum Coherence) NMR spectra of **1** showed a *meta*-coupled pair of aromatic protons (δ_H_ 7.25 and 6.83), four olefinic protons (δ_H_ 6.37, 6.04, 5.78, and 5.67), one proton attached to a dioxygenated carbon (δ_H_ 6.30), five protons connected to oxygen-bearing carbons (δ_H_ 5.26, 4.82, 4.75, 4.52, and 4.35), two aliphatic protons (δ_H_ 3.27 and 2.95), and two methyl signals (δ_H_ 1.63 and 1.56) along with four hydroxy protons (δ_H_ 11.07, 7.07, 6.70, and 6.87). The ^13^C NMR spectrum revealed ten double-bond carbons, including four quaternary carbons between 154.1 and 130.7 ppm, one dioxygenated carbon at δ_C_ 101.5, five oxygenated *sp*^3^ carbons from 84.5 to 71.4 ppm, an aliphatic carbon at δ_C_ 37.8, and two methyl carbons (δ_C_ 18.9 and 18.5). Further analysis of the ^1^H-^1^H COSY (Correlation Spectroscopy) NMR spectrum was useful for the construction of two separate spin systems. First, a diene-bearing system was elucidated by the consecutive COSY correlations from H_2_-7 (δ_C_ 3.27 and 2.95) to H_3_-13 (δ_C_ 1.63) through H-8, H-9, H-10, H-11, and H-12 (Figure 2). The geometries of the diene were established as 9*E* and 11*E* based on the *trans*-coupling constants (15.0 Hz each) of the double-bond ^1^H peaks corresponding to H-9, H-10, H-11, and H-12. The second spin system indicated the presence of a hexose. The proton bound to a dioxygenated carbon (H-1′; δ_H_ 6.30-δ_C_ 101.5) was correlated with the carbinol proton H-2′ (δ_H_ 4.82) in the COSY spectrum, revealing C-1′-C-2′ connectivity. H-2′ displayed a homonuclear coupling with H-3′ (δ_H_ 4.75), placing C-3′ next to C-2′. The COSY correlation between H-3′ and H-4′ (δ_H_ 4.35) connected C-3′ and C-4′. Further extension of the spin system was accomplished using the COSY correlations between H-4′ and H-5′ (δ_H_ 4.52) and between H-5′ and H_3_-6′ (δ_H_ 1.56), suggesting a hexose. The hydroxy protons (δ_H_ 7.07, 6.70, and 6.87) belonging to the hexose were assigned based on COSY correlations.

**Table 1:** NMR data for amycofuran (**1**) in pyridine-*d*_5_.

C/H	δ_H_^a^	Mult (*J* in Hz)	δ_C_^b^	Type
1			143.2	C
2			141.5	C
3	7.25	d (1.5)	106.9	CH
4			154.1	C
4-OH	11.07			OH
5	6.83	d (1.5)	107.7	CH
6			130.7	C
7a	3.27	dd (16.0, 8.0)	37.8	CH_2_
7b	2.95	dd (16.0, 8.0)		
8	5.26	ddd (8.0, 8.0, 8.0)	84.5	CH
9	5.78	dd (15.0, 8.0)	130.8	CH
10	6.37	dd (15.0, 10.5)	133.1	CH
11	6.04	dd (15.0, 10.5)	131.7	CH
12	5.67	dq (15.0, 7.0)	131.4	CH
13	1.63	d (7.0)	18.5	CH_3_
1′	6.30	d (1.0)	101.5	CH
2′	4.82	br m	72.5	CH
2′-OH	7.07	br s		OH
3′	4.75	br d (9.0)	72.9	CH
3′-OH	6.70	br s		OH
4′	4.35	br m	74.2	CH
4′-OH	6.87	br s		OH
5′	4.52	m	71.4	CH
6′	1.56	d (6.0)	18.9	CH_3_

^a^ 600 MHz; ^b^ 125 MHz.

The analysis of the HMBC (Heteronuclear Multiple Bond Correlation) correlations allowed complete elucidation of the planar structure of amycofuran (**1**). The long-range heteronuclear coupling from H-5’ to the dioxygenated carbon at 101.5 ppm confirmed the identity of the sugar unit as a six-membered ring. The HMBC correlations from the *meta*-coupled aromatic protons (H-3 at δ_H_ 7.25; H-5 at δ_H_ 6.83) to the aromatic carbons C-1 to C-6 established a six-membered aromatic ring. The connectivity of the diene-bearing spin system to the aromatic ring was confirmed by the HMBC correlations from H_2_-7 to C-1, C-5, and C-6. An additional long-range coupling from H-8 to C-1 indicated that the dihydrofuran ring was attached to the aromatic ring. The HMBC correlation from H-1′ to C-2 connected the sugar moiety to C-2, completing the planar structure of amycofuran (**1**) and indicating a new benzofuran.

**Figure 3:**
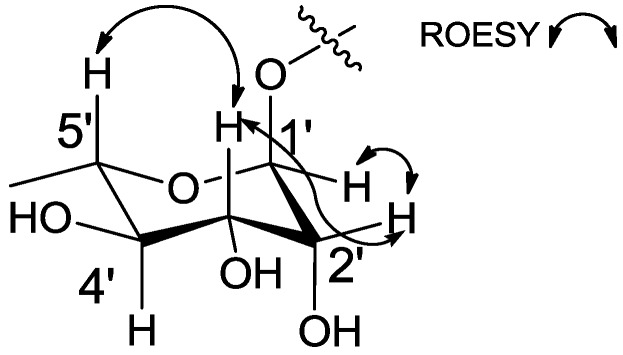
Key ROESY correlations of the hexose of **1**.

The relative configuration of the hexose was examined using the ^1^*J*_CH_ value of the anomeric proton and the ^3^*J*_HH_ values and ROESY (Rotating-frame Overhauser Effect Spectroscopy) correlations of the protons in the six-membered ring. The magnitude of the ^1^*J*_CH_ value (171 Hz) clearly revealed the α-configuration [15], placing the anomeric proton in an equatorial position. The equatorial position of H-2′ was assigned by the ROESY correlation between H-2′ and H-3′. The large ^1^H-^1^H coupling constant (9.0 Hz) between H-3′ and H-4′ established their *anti*-relationship. The 1,3-ROESY correlation between H-3′ and H-5′ established the axial position of H-5′ (Figure 3). The analysis of the relative configuration of the sugar showed that it was a rhamnose moiety, which exists solely in the l-form in nature.

**Figure 4:**
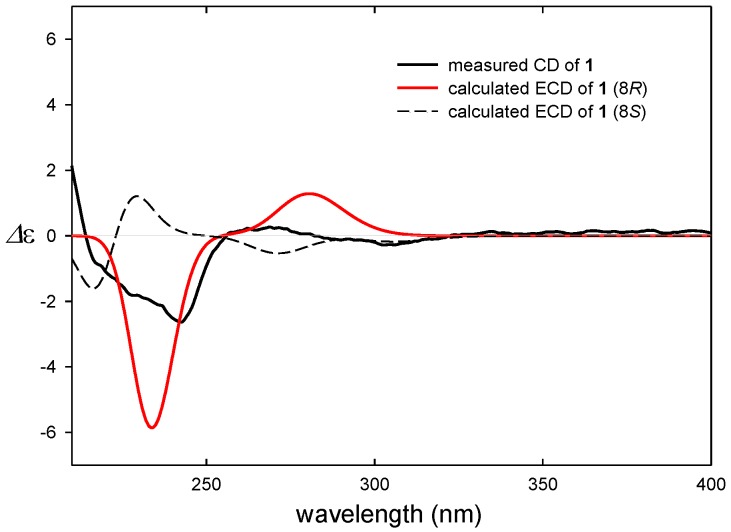
Measured CD and calculated ECD spectra of **1**.

We tried to determine the absolute configuration of C-8 in amycofuran (**1**) comparing the experimental CD (Circular Dichroism) spectrum with the calculated ECD (Electronic Circular Dichroism) spectra. Initially, because of the glycosidic bond between rhamnose and benzofuran in **1**, which can cause various changes in conformation during energy-minimized structure calculation, we carefully analyzed ROESY correlations between the sugar and aglycone to obtain a most probable conformation (see Supplementary Information). After the calculation of the energy-minimized structure from the conformation based on the ROESY correlation, we calculated ECD spectra [16,17]. The ECD spectra based on the conformation were calculated for the 8*S* and 8*R* configurations. The ECD spectrum for 8*R* turned out to be consistent with the observed CD spectrum, proposing the 8*R* configuration in amycofuran (Figure 4). In literature, a fungal metabolite asperfuran, which is aglycone of **1**, also displayed a very similar CD spectrum to that of **1**, also supporting the 8*R* configuration (see Supplementary Information) [18].

Amycocyclopiazonic acid (**2**) was purified as a white powder. Its molecular formula was determined to be C_18_H_20_N_2_O_2_ based on high-resolution mass spectrometry data as well as ^1^H and ^13^C NMR spectroscopic data (Table 2). The UV spectrum of **2** displayed characteristic indole UV absorption maxima at 222 and 280 nm. The ^1^H NMR spectrum showed one downfield singlet proton (δ_H_ 11.79) attached to a heteroatom and four aromatic resonances between 7.46 and 7.08 ppm. Two protons bound to heteroatom-bearing carbons were observed at δ_H_ 4.83 and 4.18. In addition, the ^1^H NMR spectrum displayed six aliphatic protons between 3.76 and 2.47 ppm and two methyl singlets in the upfield region (δ_H_ 1.74 and 1.65). The ^13^C and HSQC NMR spectra exhibited one carbonyl carbon (δ_C_ 170.4); eight double-bond signals from 134.5 to 109.0 ppm, including four methines and four quaternary carbons; two heteroatom-bearing *sp*^3^ methine carbons at δ_H_ 73.9 and 73.6; one quaternary carbon at δ_C_ 60.6; two aliphatic methines at δ_C_ 53.2 and 39.7; two aliphatic methylenes at δ_C_ 46.8 and 27.0; and two methyl groups at 26.4 and 22.4 ppm.

**Table 2:** NMR data for amycocyclopiazonic acid (**2**) in pyridine-*d*_5_.

C/H	δ_H_^a^	Mult (*J* in Hz)	δ_C_^b^	Type
1	11.79			NH
2	7.46	d (2.0)	120.8	CH
3			111.1	C
4	3.76	dd (10.0, 6.0)	39.7	CH
5	4.18	dd (10.0, 7.0)	73.6	CH
6	4.83	ddd (7.0, 7.0, 6.0)	73.9	CH
7	2.99	m	46.8	CH_2_
8			170.4	C
9				N
10			60.6	C
11	2.47	m	53.2	CH
12a	3.13	dd (12.0, 6.0)	27.0	CH_2_
12b	3.03	dd (12.0, 6.0)		
13			129.7	C
14	7.08	d (7.5)	116.1	CH
15	7.34	dd (8.0, 7.5)	122.4	CH
16	7.43	d (8.0)	109.0	CH
17			134.5	C
18			126.9	C
19	1.65	s	22.4	CH_3_
20	1.74	s	26.4	CH_3_

^a^ 600 MHz; ^b^ 125 MHz.

An analysis of the COSY and HMBC correlations established the expected indole moiety. Next, the COSY correlations and ^1^H-^1^H coupling constants (7.5–8.0 Hz) of the double-bond protons H-14 (δ_H_ 7.08), H-15 (δ_H_ 7.34), and H-16 (δ_H_ 7.43) connected the three consecutive carbons (C-14, C-15, and C-16) in a six-membered aromatic ring. The HMBC correlations from the three aromatic protons (H-14, H-15, and H-16) and an additional aromatic singlet proton to aromatic carbons revealed an indole substructure (Figure 2). The C-12–C-11 connectivity was determined from the COSY correlations between H_2_-12 (δ_H_ 3.13 and 3.03) and H-11 (δ_H_ 2.47). C-11 was then connected to C-4 by the homonuclear coupling between H-11 and H-4 (δ_H_ 3.76). The spin system was further extended to C-5, C-6, and C-7 by an array of COSY correlations involving H-5, H-6, and H_2_-7. The HMBC correlation from H_2_-7 to C-8 (δ_C_ 170.4) extended the connection to the carbonyl carbon, which was verified as an amide carbon based on the IR absorption at 1671 cm^−1^. HMBC cross peaks from the methyl groups (δ_H_ 1.65 and 1.74) to C-10, C-11, C-19, and C-20 indicated a *gem*-dimethyl moiety connected to C-11. The carbon chemical shift of C-10 (δ_C_ 60.6) indicated that this carbon bears a nitrogen atom, placing the nitrogen atom of the amide functional group next to C-10. Finally, the connectivity from C-5 to the amide nitrogen atom was deduced by the carbon chemical shift of C-5 (δ_C_ 73.6) and the degree of unsaturation. Therefore, the planar structure of **2** was elucidated, and it was determined to be a new member of the indole alkaloid cyclopiazonic acid class.

**Figure 5:**
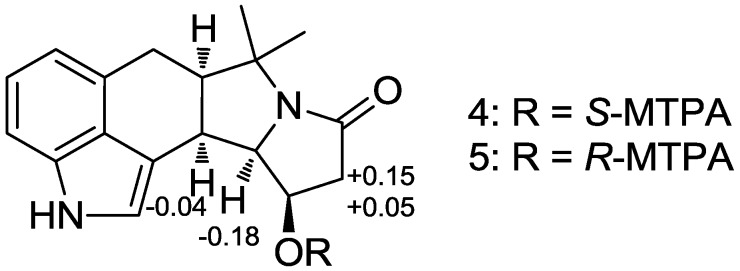
Δδ*_S_*_−*R*_ values of **4** and **5** in pyridine-*d*_5_.

The relative stereochemistry of **2** was readily assigned by ROESY NMR spectroscopic analysis. Strong ROESY correlations among H-4, H-5, and H-6 revealed that these protons are on the same side. The ROESY correlation between H-4 and H-11 established their *syn*-relationship, thus determining the entire relative configuration of **2**. The absolute configuration of amycocyclopiazonic acid was assigned by the modified Mosher method [19]. The esterification of **2** with *R*- and *S*-MTPA-Cl (α-methoxy-α-(trifluoromethyl) phenylacetyl chloride) yielded the *S*- and *R*-MTPA esters of **2** (**4** and **5**, respectively). The analysis of the ^1^H NMR Δδ*_S-R_* values revealed a clearly consistent sign distribution, thus confirming the absolute configuration of **2** as 4*R*, 5*R*, 6*R,* and 11*R* (Figure 5).

Amycolactam (**3**) was obtained as a white powder with a molecular formula of C_18_H_22_N_2_O_3_, as determined by HR-ESI-MS and ^1^H and ^13^C NMR spectroscopic data (Table 3). The UV spectrum of **3** was very similar to that of **2**, indicating that this compound also possesses an indole substructure. The ^1^H NMR spectrum showed three downfield protons attached to a heteroatom (δ_H_ 12.04, 8.87, and 6.78), four aromatic protons from 7.61 to 7.13 ppm, one olefinic proton at δ_H_ 5.56, three protons bound to carbons bearing a nitrogen or oxygen atom (δ_H_ 4.76, 4.73, and 4.26), four aliphatic protons (δ_H_ 4.02 (2H), 3.93, and 3.78), and two methyl groups (δ_H_ 1.70 and 1.67). The ^13^C and HSQC NMR spectra enabled the identification of one quaternary carbonyl carbon (δ_C_ 176.8); ten *sp*^2^ carbons, including five methines and five quaternary carbons between 138.7 and 110.6; two oxygen-bearing methines (δ_C_ 74.2 and 71.4); one nitrogen-bound methine (δ_C_ 57.1); two aliphatic methylenes (δ_C_ 32.7 and 27.9); and two methyl groups (δ_C_ 25.8 and 18.0).

Three partial structures were identified on the basis of the analysis of the COSY and HMBC NMR spectra. In accordance with the UV spectrum, an indole moiety was readily assigned in a manner analogous to that used for amycocyclopiazonic acid (**2**). A dihydroxy γ-lactam ring was indicated by the COSY correlation from H-6 (δ_H_ 4.73) to H-5 (δ_H_ 4.26) and H-7 (δ_H_ 4.76) as well as the long-range heteronuclear coupling from H-5 and H-7 to the amide carbon C-8 (δ_C_ 176.8). An additional COSY correlation between H-5 and H_2_-4 (δ_H_ 3.93 and 3.78) connected the lactam ring to the C-4 methylene. The HMBC correlation from the methyl group protons H_3_-19 (δ_H_ 1.67) and H_3_-20 (δ_H_ 1.70) to C-10 (δ_C_ 131.9) and C-11 (δ_C_ 125.1) constructed a dimethyl group bonded to an alkene carbon. The double bond was connected to an aliphatic methylene C-12 (δ_C_ 32.7) by the ^1^H-^1^H coupling between H-11 (δ_H_ 5.56) and H_2_-12 (δ_H_ 4.02), allowing the assignment of an isoprene unit. These three partial structures were assembled by heteronuclear correlations. The HMBC coupling from H_2_-4 to C-3 (δ_C_ 112.6) connected the dihydroxy γ-lactam ring to the indole ring. Furthermore, the two-bond heteronuclear correlation from H_2_-12 to C-13 placed the isoprene unit next to C-13 (δ_C_ 134.8), finalizing the planar structure of amycolactam (**3**) and indicating that it is a new indole alkaloid bearing an isoprene unit and a dihydroxy γ-lactam (Figure 2).

**Table 3:** NMR data for amycolactam (**3**) in pyridine-*d*_5_.

C/H	δ_H_^a^	Mult (*J* in Hz)	δ_C_^b^	Type
1	12.04	s		NH
2	7.61	d (2.0)	124.9	CH
3			112.6	C
4a	3.93	dd(15.0, 7.5)	27.9	CH_2_
4b	3.78	dd(15.0, 7.5)		
5	4.26	m	57.1	CH
6	4.73	dd (5.0, 3.5)	71.4	CH
6-OH	6.78			OH
7	4.76	d (5.0)	74.2	CH
7-OH	8.87			OH
8			176.8	C
9	ND ^c^			NH
10			131.9	C
11	5.56	dd (6.0, 6.0)	125.1	CH
12	4.02	m	32.7	CH_2_
13			134.8	C
14	7.13	d (7.0)	120.1	CH
15	7.29	dd (8.0, 7.0)	122.4	CH
16	7.53	d (8.0)	110.6	CH
17			138.7	C
18			126.4	C
19	1.67	s	25.8	CH_3_
20	1.70	s	18.0	CH_3_

^a^ 600 MHz; ^b^ 125 MHz; ^c^ Not detected.

**Figure 6:**
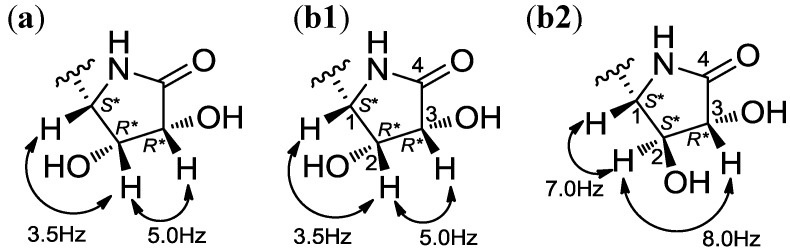
(**a**) ^1^H-^1^H coupling constants of the dihydroxy γ-lactam ring observed in **3**. ^1^H-^1^H coupling constants of (**b1**) the dihydroxy γ-lactam ring possessing 1S*, 2R*, and 3R*, and (**b2**) 1S*, 2S*, and 3R* found in literature.

The relative configuration of the dihydroxy γ-lactam ring was firstly deduced from the ROESY correlations and ^1^H–^1^H coupling constants. A clear correlation between H-5 and H-7 led to the well supported assignment of a *syn*-relationship between these two protons. However, the chemical shifts of H-6 (δ_H_ 4.73) and H-7 (δ_H_ 4.76) were too close to allow the definitive assignment of the relative configuration of C-6 from the ROESY correlation. Thus, we analyzed the ^1^H–^1^H coupling constants in the dihydroxy γ-lactam ring by comparing them with literature values (Figure 6). The coupling constants ^3^*J*_H5H6_ and ^3^*J*_H6H7_ in **3** were 5.0 and 3.5 Hz, respectively. These values are consistent with the coupling constants of a dihydroxy γ-lactam ring with the S*, R*, and R* configurations, whereas an alternative isomer with the S*, S*, and R* configurations showed significantly different coupling constants [20,21,22].

To determine the absolute configuration, we first attempted to use the modified Mosher method, but amycolactam (**3**) decomposed during MTPA esterification, even under mild conditions. Therefore, ECD calculation was utilized to determine the absolute configuration of **3**. When we performed the ECD calculation for **3**, we found that the existence of two or more conformations. Then, we optimized the conformers of **3** using the basis set def-SV(P) for all atoms and the functional B3LYP/DFT level and considered the conformers when calculating the ECD spectra with TDDFT using the basis set def2-TZVPP for all atoms and the functional B3LYP/DFT level. The calculated ECD spectra of (5*S*, 6*R*, 7*R*)-amycolactam (**3**) displayed negative Cotton effects at 242 nm, consistent with the experimental CD spectrum (see the Supplementary Information).

The structure of amycofuran (**1**), which is a benzofuran glycoside, is interesting. To the best of our knowledge, no benzofuran substituted together with a diene moiety and a rhamnose sugar has been previously reported. A comprehensive literature search indicated that benzofurans are very rare as bacterial secondary metabolites, although a few examples, such as actiketal [23] and isoaurostatin [24], have been reported from actinomycetes whereas benzofurans are relatively common in fungi [18,25]. Amycocylopiazonic acid (**2**) is a new member of the cyclopiazonic acid class, which was previously reported to be produced by fungi belonging to the genus *Penicillium* [26]. Interestingly, this class has not been discovered from a bacterium. Therefore, amycocyclopiazonic acid (**2**) is the first bacterially produced member of the cyclopiazonic acid class. Based on the carbon backbone structure of amycolactam (**3**), this new alkaloid may be considered as an acyclic version of amycocyclopiazonic acid (**2**) generated during cyclopiazonic acid biosynthesis [27]. The biological activities of **1**–**3** were evaluated in antibacterial and antifungal assays against various pathogenic microbes, but the compounds did not exhibit significant inhibitory activities. The cytotoxicities of **1**–**3** were tested against human carcinoma cell lines, including A549 (lung cancer), HCT116 (colon cancer), SNU638 (gastric cancer), K562 (leukemia), SK-HEP1 (liver cancer), and MDA-MB231 (breast cancer). Amycolactam (**3**) significantly inhibited the proliferation of SNU638 and HCT116, with IC_50_ values of 0.8 and 2.0 μM, respectively. The cytotoxic effects of **3** against A546, K562, and SK-HEP1 were moderate (IC_50_ = 13.7, 9.6, and 8.3 μM, respectively), whereas virtually no cytotoxicity was observed against MDA-MB231. However, amycofuran (**1**) and amycocyclopiazonic acid (**2**) did not exhibit significant cytotoxicity against the cancer cells tested.

## 3. Experimental Section

### 3.1. General Experimental Procedures

Optical rotations were obtained in a 1 cm cell with a Jasco P-1020 polarimeter. UV spectra were measured with a Perkin Elmer Lambda 35 UV/VIS spectrometer. IR spectra were recorded with a Thermo N1COLET iS10 spectrometer. A Bruker Avance 600 MHz spectrometer at NCIRF (National Center for Inter-University Research Facilities, Seoul National University, Seoul, Korea) was used to obtain ^1^H, ^13^C, and 2D NMR spectra. Electrospray ionization (ESI) low-resolution LC/MS data were collected on an Agilent Technologies 6130 Quadrupole mass spectrometer attached to an Agilent Technologies 1200 series HPLC using a reversed-phase C_18_ column (Phenomenex Luna, 100 × 4.6 mm, Torrance, CA, USA). High-resolution electrospray ionization (HR-ESI) mass spectra were acquired with a Thermo Finnigan LCQ high-resolution mass spectrometer at NICEM (National Instrumentation Center for Environmental Management, Seoul National University, Seoul, Korea). Circular Dichroism was measured with an Applied Photophysics Chirascan plus (Surrey, UK) with a 0.2 cm cell at NICEM.

### 3.2. Isolation of Bacteria, Cultivation, and Extraction

A sponge sample was gathered from Micronesia. One gram of unidentified sponge sample was extracted with 3 mL of sterilized artificial seawater at a 1:3 dilution ratio and shaken at 180 rpm for 1 h. A total of 150 μL of the resulting solution was spread on Gause1 agar (20 g soluble starch, 1 g KNO_3_, 0.5 g K_2_HPO_4_, 0.01 g FeSO_4_·7H_2_O), M2 agar (6 mL glycerol, 1 g arginine, 1 g KH_2_PO_4_, 0.5 g MgSO_4_·7H_2_O, 16.0 g agar), SC agar (10.0 g starch, 0.3 g casein, 2.0 g KNO_3_, 2.0 g K_2_HPO_4_, 0.05 g MgSO_4_·7H_2_O, 0.02 g CaCO_3_, 0.01 g FeSO_4_·7H_2_O), IM6 agar (0.5 g glycerol, 0.5 g starch, 0.5 g sodium propionate, 0.1 g KNO_3_, 0.1 g asparagine, 0.3 g casein, 0.5 g K_2_HPO_4_, 1 mg FeSO_4_·7H_2_O, 16.0 g agar, 1 mg vitamin B), and K agar (25 g soluble starch, 15 g soy peptone, 2 g dry yeast, 4 g CaCO_3_). The strain Cra33g was isolated on Gause1 agar. An analysis of the 16S rDNA sequence (Figure S23 in the Supplementary Information) indicated that Cra33g is most similar to *Amycolatopsis saalfeldensis*. The strain Cra33g was cultivated in 60 mL S medium (25 g soluble starch, 15 g soy peptone, 2 g dry yeast, 4 g calcium carbonate in 1 L artificial seawater) in a 150 mL Erlenmeyer flask. After cultivation for 70 h on a rotary shaker at 180 rpm at 27 °C, a 5 mL aliquot of the culture was transferred into 250 mL Diaion LH-20 (1 g/L) with GSS medium (10 g soluble starch, 20 g glucose, 25 g soy peptone, 1 g beef extract, 4 g yeast extract, 2 g NaCl, 0.25 g KH_2_PO_4_, and 2 g CaCO_3_ in 1 L artificial seawater) in 500 mL Erlenmeyer flasks (20 ea × 250 mL; total volume 5 L). After 10 days of cultivation, 18 L of ethyl acetate was added to the whole culture (5 L) and shaken for extraction. Anhydrous sodium sulfate was added to the separated organic layer, which was then filtered and dried. In total, the ethyl acetate extract yielded 4.5 g (a rotary evaporator was used to concentrate the extracts *in vacuo*). This procedure was performed 10 times (50 L culture; total amount of extract: 45 g) to acquire quantities of **1**–**3** sufficient for structure elucidation and bioassays.

### 3.3. Isolation of **1**–**3**

The dried ethyl acetate crude extract was adsorbed on Celite and then loaded on a 6 g Sep-Pak C_18_ open column and fractionated with 100 mL each of 20%, 40%, 60%, 80%, and 100% MeOH in water and 1:1 MeOH/dichloromethane. The 40%, 60%, and 80% MeOH/water fractions contained **1**–**3**. The combined 40%, 60%, and 80% MeOH/water fractions were eluted on silica gel and a Sephadex LH-20 column to remove the fatty acids, which would have HPLC (high-performance liquid chromatography) retention times similar to **1**–**3** in the subsequent HPLC purification. Reversed-phase HPLC (Kromasil C_18_ (2): 250 × 10 mm; 5 μm) under isocratic conditions in 4:6 acetonitrile/water (UV 220 nm detection; flow rate: 2 mL/min) was used to purify **1**–**3** from the three fractions. Finally, amycofuran (**1**) (20 mg), amycocyclopiazonic acid (**2**) (18 mg), and amycolactam (**3**) (10 mg) were obtained at the retention times of 12 min, 13 min, and 15 min, respectively.

#### 3.3.1. Amycofuran (**1**)

[α]_D_ −2 (c 0.10, MeOH); UV (MeOH) *λ*_max_ (log ε) 214 (3.76), 227 (3.89), 297 (2.96) nm; CD (MeOH) (Δε) 243 (−2.61), 257 (0.10), 269 (0.27), 283 (−0.09) nm; IR (neat) *ν*_max_ 2980, 2971, 1605, 1455, 1346, 1032 cm^−1^; for ^1^H and ^13^C NMR data, see Table 1; HRESIMS at *m/z* 387.1407 [M + Na]^+^ (calcd for C_19_H_24_O_7_Na 387.1420).

#### 3.3.2. Amycocyclopiazonic acid (**2**)

[α]_D_ −16 (c 0.10, MeOH); UV (MeOH) *λ*_max_ (log ε) 207 (2.84), 222 (3.02), 280 (2.29) nm; IR (neat) ν_max_ 3309, 2980, 1671, 1413 cm*^−^*^1^; for ^1^H and ^13^C NMR data, see Table 2; HRESIMS at *m/z* 297.1591 [M + H]^+^ (calcd for C_18_H_21_N_2_O_2_ 297.1603).

#### 3.3.3. Amycolactam (**3**)

[α]_D_ −3 (c 0.05, MeOH); UV (MeOH) *λ*_max_ (log ε) 214 (3.07), 221 (3.08), 275 (2.49) nm; CD (MeOH) (Δε) 211 (0.51), 267 (−1.56) nm; IR (neat) *ν*_max_ 3402, 2971, 2921, 1687, 1032 cm^−1^; for ^1^H and ^13^C NMR data, see Table 3; HRESIMS at *m/z* 315.1694 [M + H]^+^ (calcd for C_18_H_23_N_2_O_3_ 315.1709).

### 3.4. MTPA Esterification of Amycocyclopiazonic Acid (**2**)

Two 40 mL vials containing **2** (1 mg sample in each vial) were completely dried under high vacuum for 10 h. Freshly distilled anhydrous pyridine (1 mL) and catalytic amounts of crystalline dimethylaminopyridine (DMAP) were added to each reaction vial in that order under argon gas. Then, *R*- and *S*-α-methoxy trifluoromethyl-phenylacetic acid (MTPA) chloride (20 μL of *R*-MTPA-Cl and 60 μL of *S*-MTPA-Cl) were separately added. Whereas the reaction with *R*-MTPA chloride was completed at room temperature after 90 min, the reaction with *S*-MTPA chloride required 2 h at 50 °C. The reactions were quenched by the addition of 50 μL of MeOH. The products were purified using reversed-phase HPLC (Kromasil C_18_ (2): 250 × 10 mm; 5 μm) with a gradient (40% to 100% aqueous acetonitrile for 30 min and followed by 100% acetonitrile under isocratic conditions for 30 min). The *S*- and *R*-MTPA esters (**4** and **5**, respectively) of **2** eluted at 45.5 and 50.4 min, respectively. The Δδ*_S-R_* values around the stereogenic centers of the MTPA esters were assigned based on the ^1^H and ^1^H-^1^H COSY NMR spectra.

#### 3.4.1. *S*-MTPA Ester (**4**) of Amycocyclopiazonic Acid (**2**)

^1^H NMR (600 MHz, pyridine-d_5_) δ 11.95 (s, 1H), 7.46 (d, *J* = 8.0, 1H), 7.38–7.31 (m, 5H), 7.09–7.05 (m, 3H), 6.04 (ddd, *J* =17.0, 8.5, 7.0, 1H), 3.99 (dd, *J* = 10.5, 7.0, 1H), 3.77 (dd, *J* = 10.5, 6.5, 1H), 3.54 (s, 3H), 3.15 (dd, *J* = 17.0, 8.5, 1H), 2.98–2.93 (m, 3H), 2.41 (dq, *J* = 12.0, 6.5, 1H), 1.67 (s, 3H), 1.60 (s, 3H). The molecular formula of **4** was confirmed as C_28_H_2__7_N_2_O_4_F_3_ ([M + H]^+^ at *m/z* 513).

#### 3.4.2. *R*-MTPA Ester (**5**) of Amycocyclopiazonic Acid (**2**)

^1^H NMR (600 MHz, pyridine-d_5_) δ 11.90 (s, 1H), 7.53 (d, *J* = 8.0, 1H), 7.46 (d, *J* = 8.0, 2H), 7.37 (d, *J* = 4.5, 1H), 7.27 (dd, *J* = 15.0, 8.0, 3H), 7.10 (d, *J* = 7.0, 1H), 7.05 (s, 1H), 6.10 (ddd, *J* = 16.0, 9.0, 7.0, 1H), 4.16 (dd, *J* = 10.5, 7.0, 1H), 3.81 (dd, *J* = 10.5, 5.5, 1H), 3.38 (s, 3H), 3.10 (m, 1H), 3.07 (dd, *J* = 16.0, 8.0, 1H), 2.99 (dd, *J* = 16.0, 8.0, 1H), 2.81 (dd, *J* = 16.0, 9.0, 1H), 2.44 (dq, *J* = 16.0, 6.5, 1H), 1.70 (s, 3H), 1.67 (s, 3H). The molecular formula of **5** was confirmed as C_28_H_2__7_N_2_O_4_F_3_ ([M + H]^+^ at *m/z* 513).

### 3.5. ECD Computational Calculation

The theoretical calculations of compound **1**, the aglycone of compound **1**, and **3** were performed using Turbomole 6.5. The optimized conformation geometries and energy levels are provided in the Supplementary Information (Tables S1–S3). The theoretical calculation of ECD was performed using TDDFT using the basis set def2-TZVPP for all atoms and the functional B3LYP/DFT level. The ECD spectra are obtained by Gaussian functions for each transition.

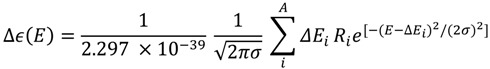
(1)
where *σ* represents the width of the band at 1/*e* height and *ΔE_i_* and *R_i_* are the excitation energies and rotational strengths for transition *i*, respectively. *σ* = 0.10 eV and *R*^vel^°^city^ were used in this work.

### 3.6. Evaluation of Anti-Proliferative Activity

Cell proliferation was measured using the sulforhodamine B (SRB) assay. Briefly, six human cancer cell lines (A549, HCT116, SNU638, K562, SK-HEP1, and MDA-MB231) (3 × 10^5^ cells/mL) were seeded in 96-well plates with various concentrations of **1**–**3** and incubated at 37 °C in a humidified atmosphere with 5% CO_2_. After 72 h of treatment with amycofuran (**1**), amycocyclopiazonic acid (**2**), and amycolactam (**3**), the cells were fixed with a 10% TCA solution for 1 h, and cellular proteins were stained with 0.4% SRB in a 1% acetic acid solution. The stained cells were dissolved in 10 mM Tris buffer (pH 10.0). The effect of **1**–**3** on cell proliferation was calculated as the percentage relative to a solvent-treated control, and the IC_50_ values were determined using nonlinear regression analysis (percent survival *versus* concentration).

## 4. Conclusions

Investigation of the secondary metabolites produced by a sponge-associated rare actinomycete, *Amycolatopsis* sp., led to the discovery of amycofuran (**1**), amycocyclopiazonic acid (**2**), and amycolactam (**3**). Amycofuran (**1**) is a structurally new benzofuran bearing a rhamnose sugar. Amycocyclopiazonic acid (**2**) and amycolactam (**3**) are the first bacterial indole alkaloids related to the cyclopiazonic acid class, which has previously only been found in fungi. Amycolactam showed significant cytotoxicity against gastric carcinoma cells (SNU638) and colon cancer cells (HCT116). Our findings indicate that the relatively underinvestigated sponge-associated rare actinomycetes have great potential for the discovery of bioactive small molecules.

## Supplementary Files

Supplementary File 1 Supplementary Information (PDF, 940 KB)
