# Supplementary material for: A New Benzofuran Glycoside and Indole Alkaloids from a Sponge-Associated Rare Actinomycete, Amycolatopsis sp
Source: Mar Drugs. 2014 Apr 22;12(4):2326–40. doi: 10.3390/md12042326 (PMC4012469; doi:10.3390/md12042326)
Supplement: Supplementary File 1 — Supplementary Information (PDF, 940 KB) [file marinedrugs-12-02326-s001.pdf]

Supplementary Information

Figure S1. ^{1}H NMR spectrum of Amycofuran (**1**) at 600 MHz in pyridine-d_5.

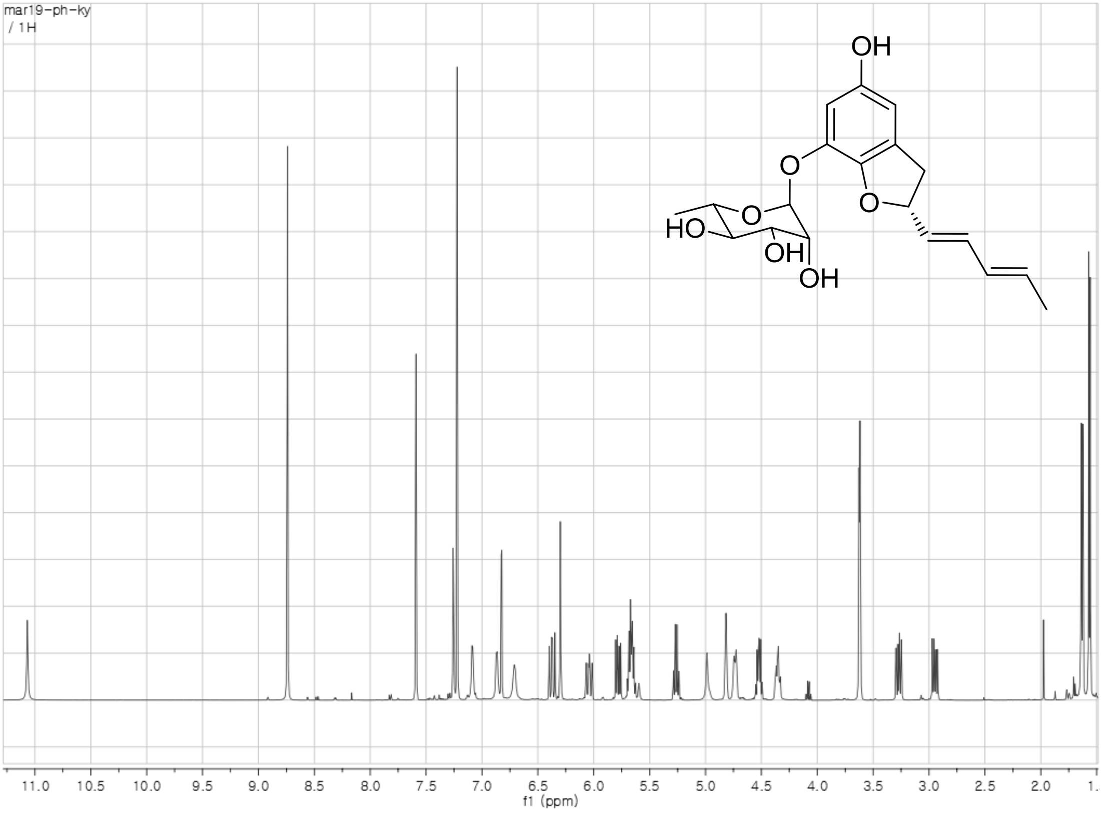

Figure S2. ^{13}C NMR spectrum of Amycofuran (**1**) at 150 MHz in pyridine-d_5.

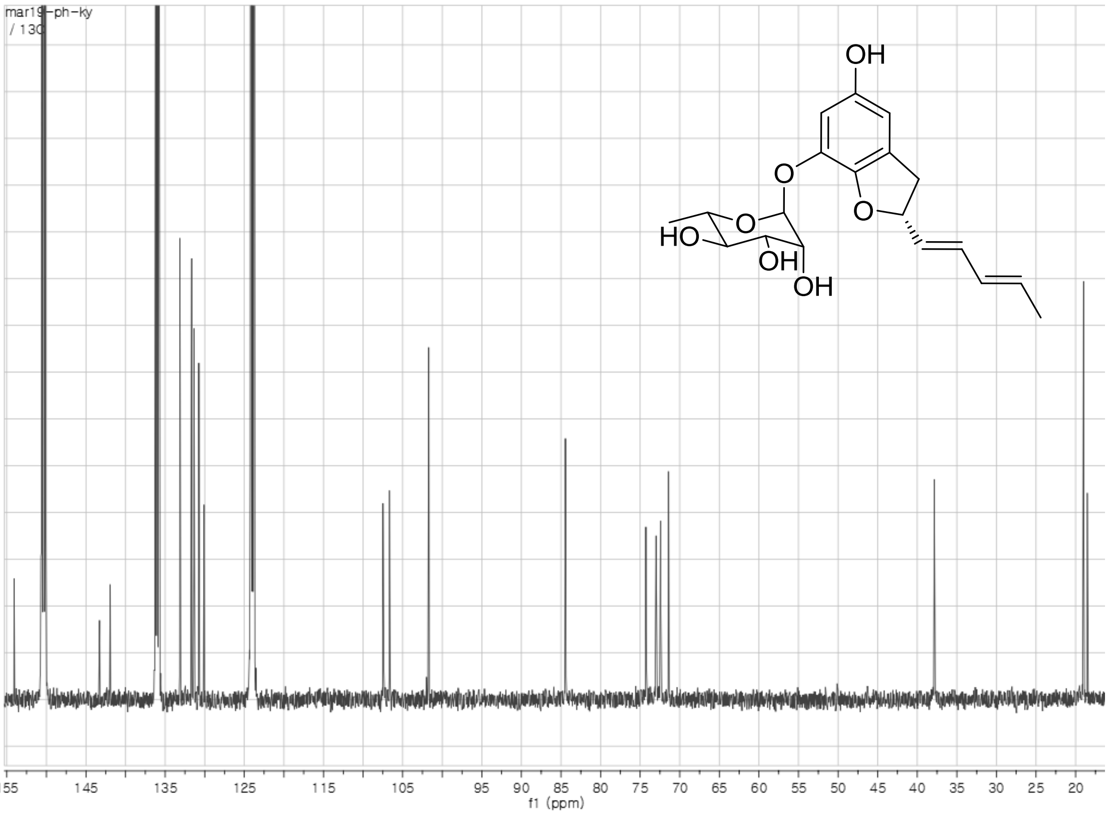

Figure S3. ^{1}H–^{1}H COSY NMR spectrum of Amycofuran (**1**) at 600 MHz in pyridine-d_5.

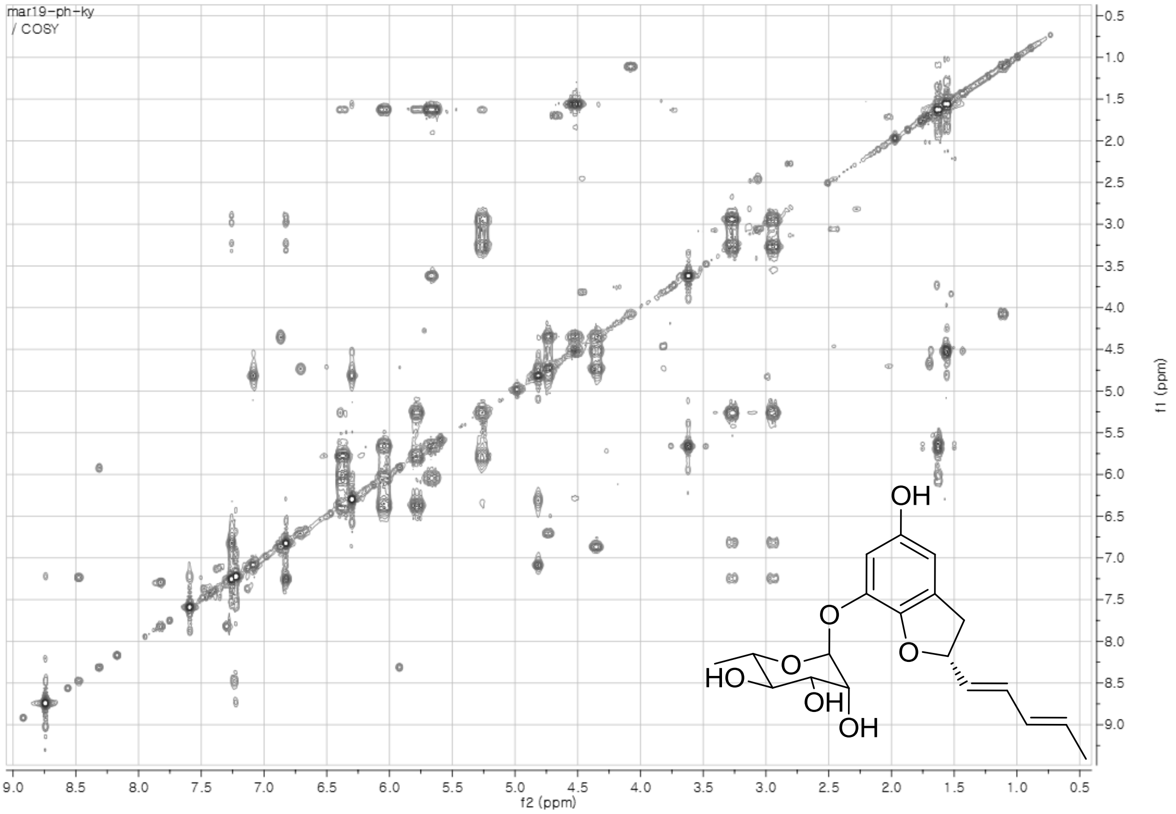

Figure S4. HSQC NMR spectrum of Amycofuran (**1**) at 600 MHz in pyridine-d_5.

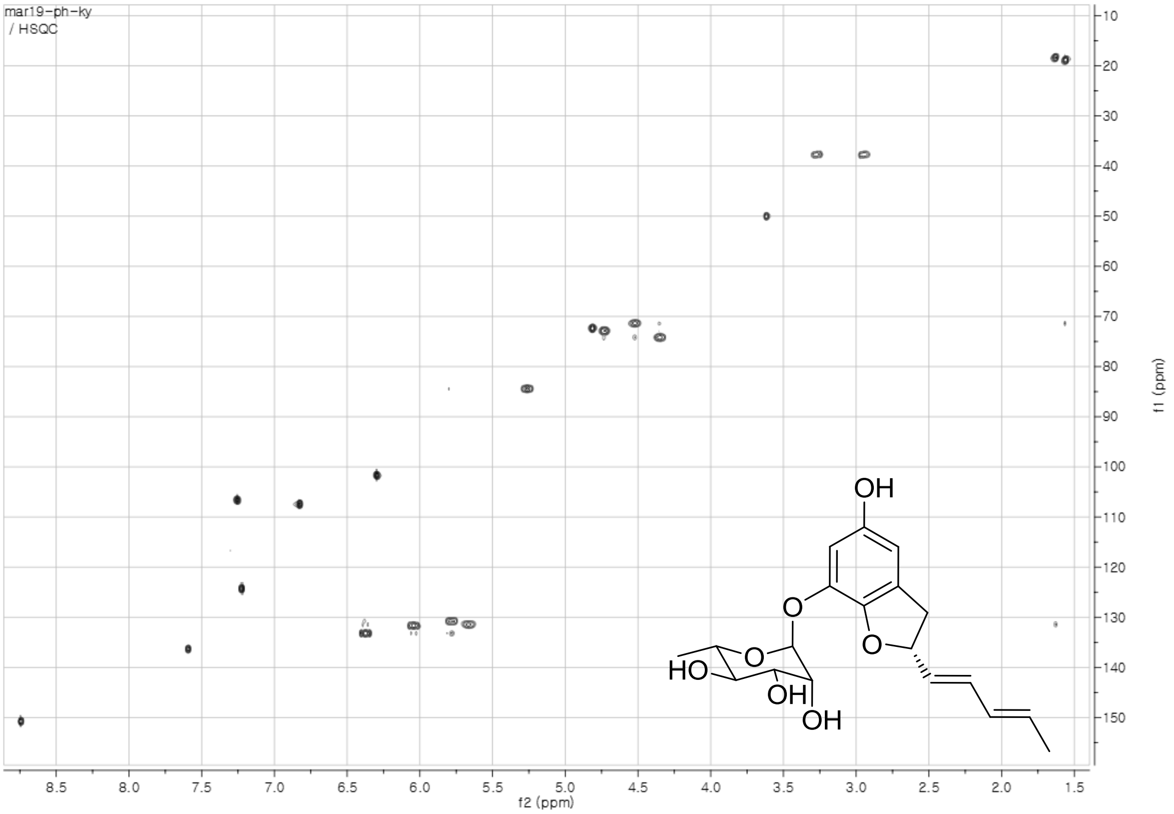

Figure S5. HMBC NMR spectrum of Amycofuran (**1**) at 600 MHz in pyridine-d_5.

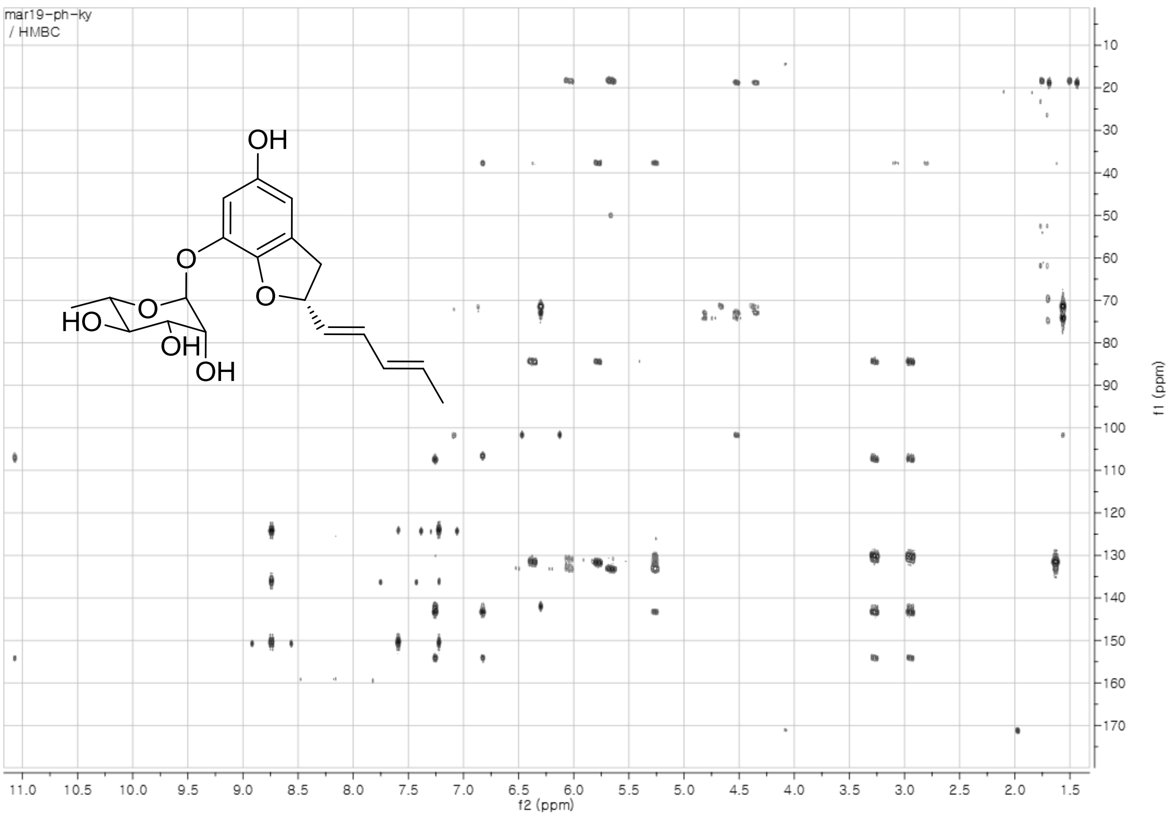

Figure S6. ^{1}H–^{1}H ROESY NMR spectrum of Amycofuran (**1**) at 600 MHz in pyridine-d_5.

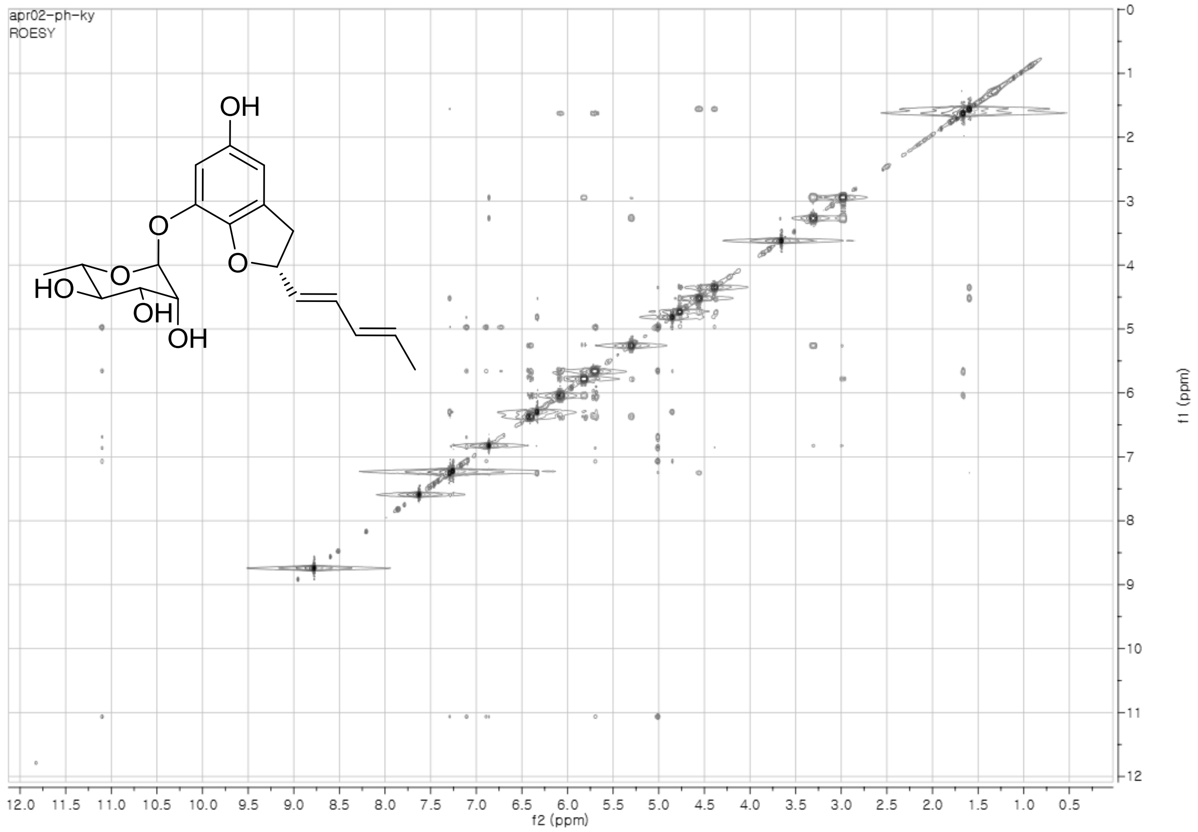

Figure S7. ^{1}H NMR spectrum of Amycocyclopiazonic acid (**2**) at 600 MHz in pyridine-d_5.

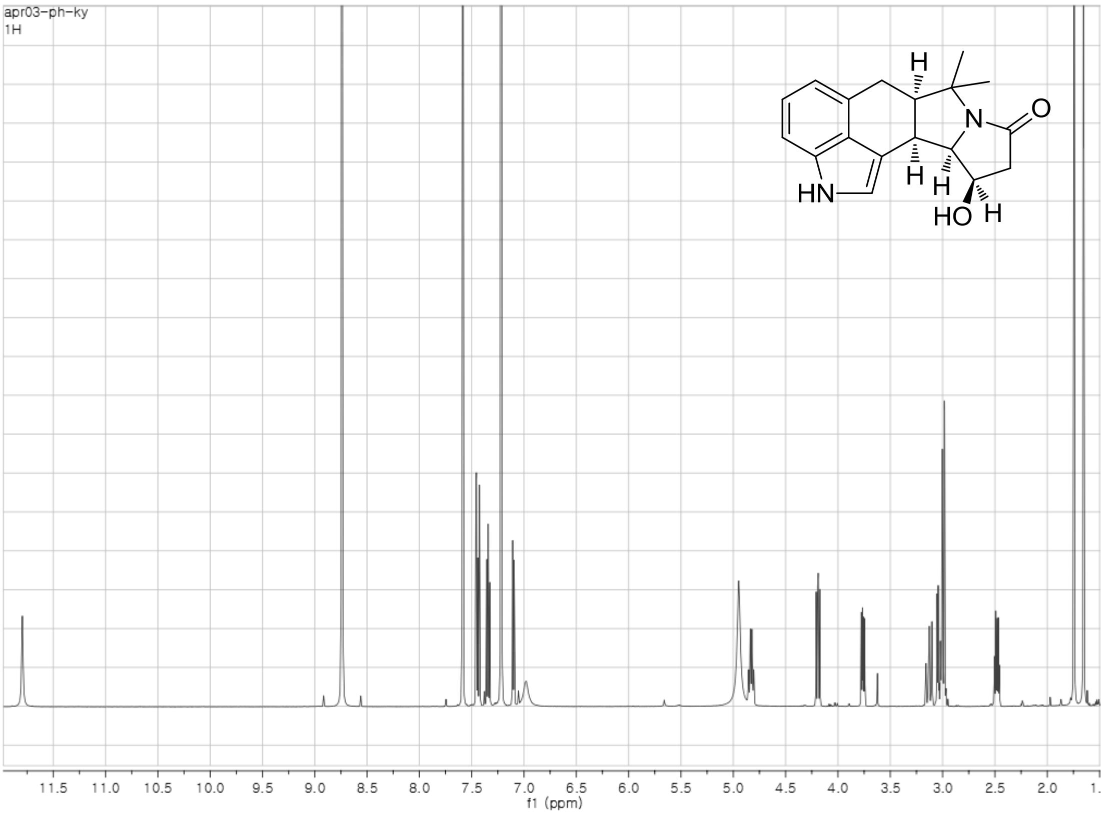

Figure S8. ^{13}C NMR spectrum of Amycocyclopiazonic acid (**2**) at 150 MHz in pyridine-d_5.

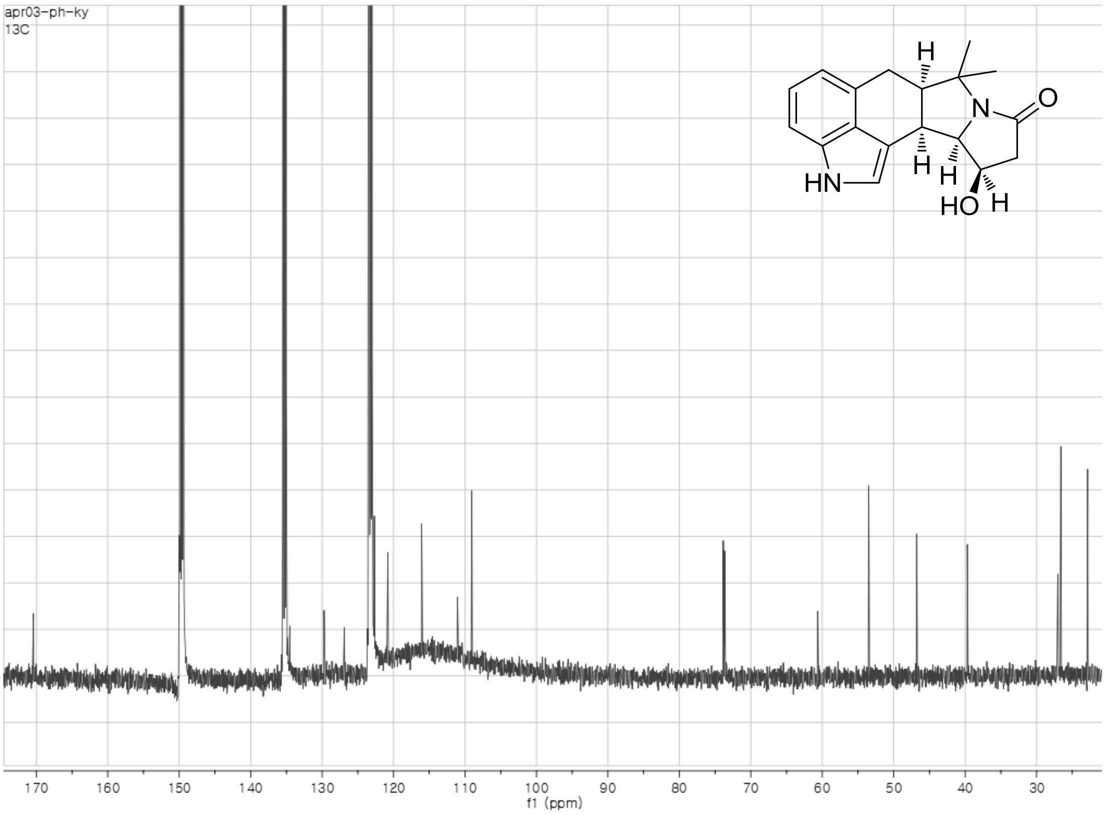

Figure S9. ^{1}H–^{1}H COSY NMR spectrum of Amycocyclopiazonic acid (**2**) at 600 MHz in pyridine-d_5.

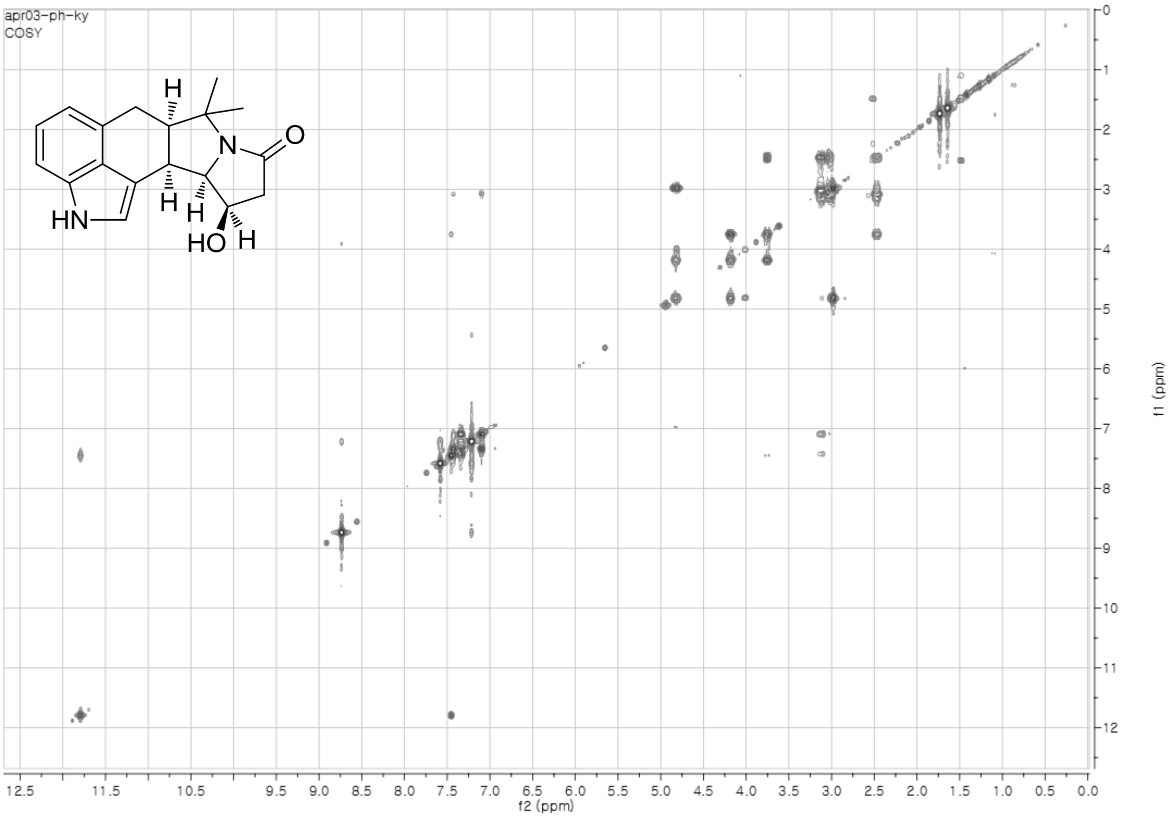

Figure S10. HSQC NMR spectrum of Amycocyclopiazonic acid (**2**) at 600 MHz in pyridine-d_5.

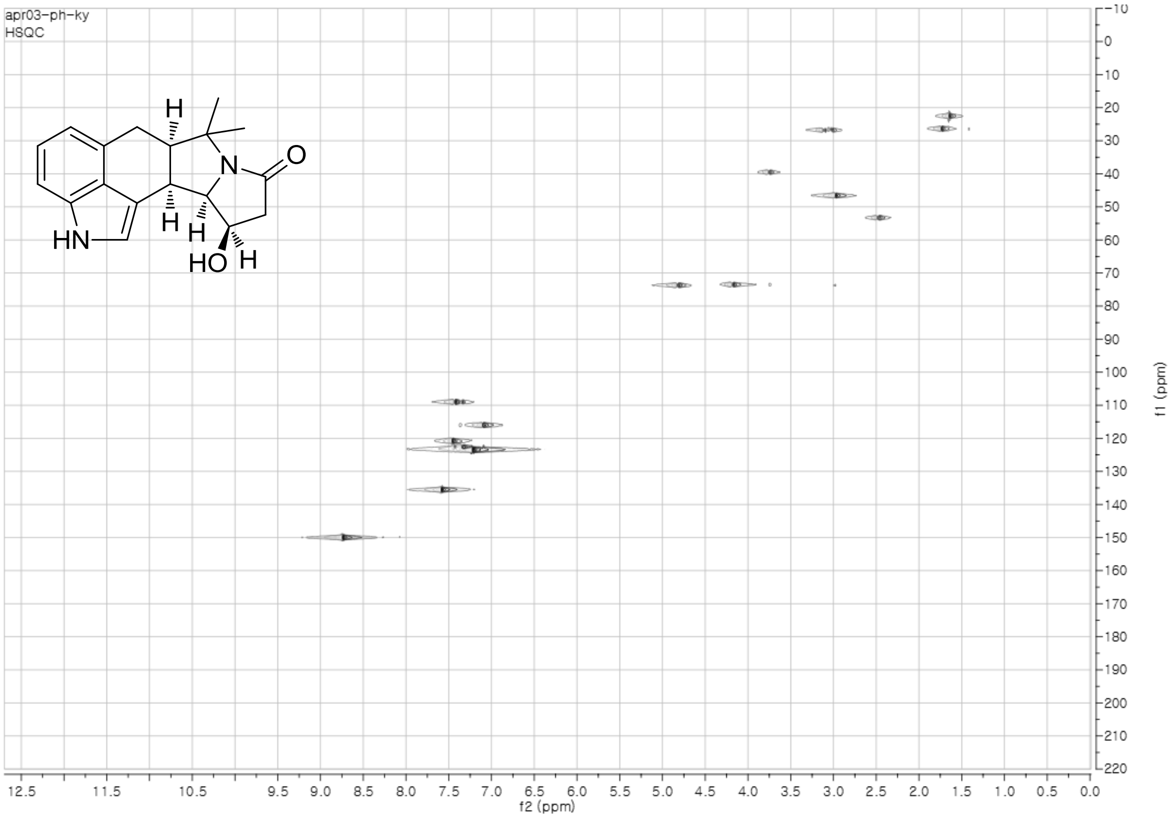

Figure S11. HMBC NMR spectrum of Amycocyclopiazonic acid (**2**) at 600 MHz in pyridine-d_5.

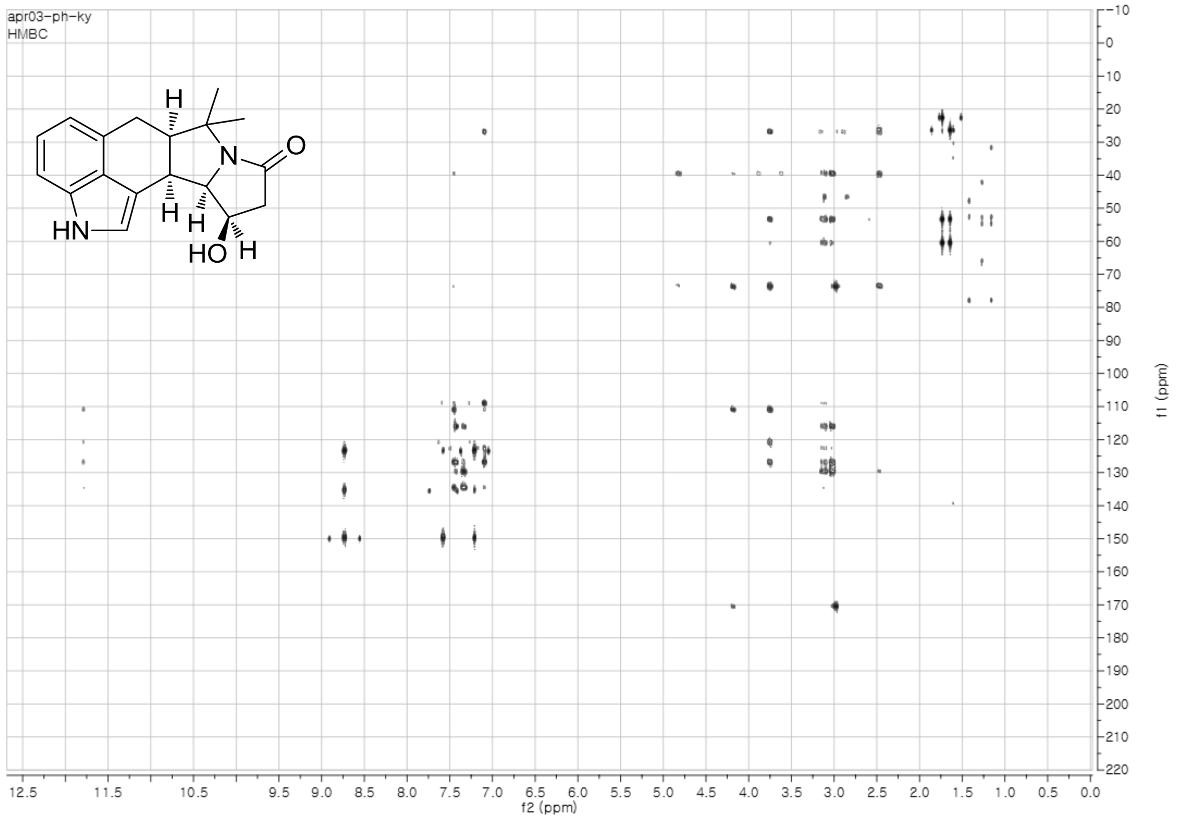

Figure S12. ^{1}H–^{1}H ROESY NMR spectrum of Amycocyclopiazonic acid (**2**) at 600 MHz in pyridine-d_5.

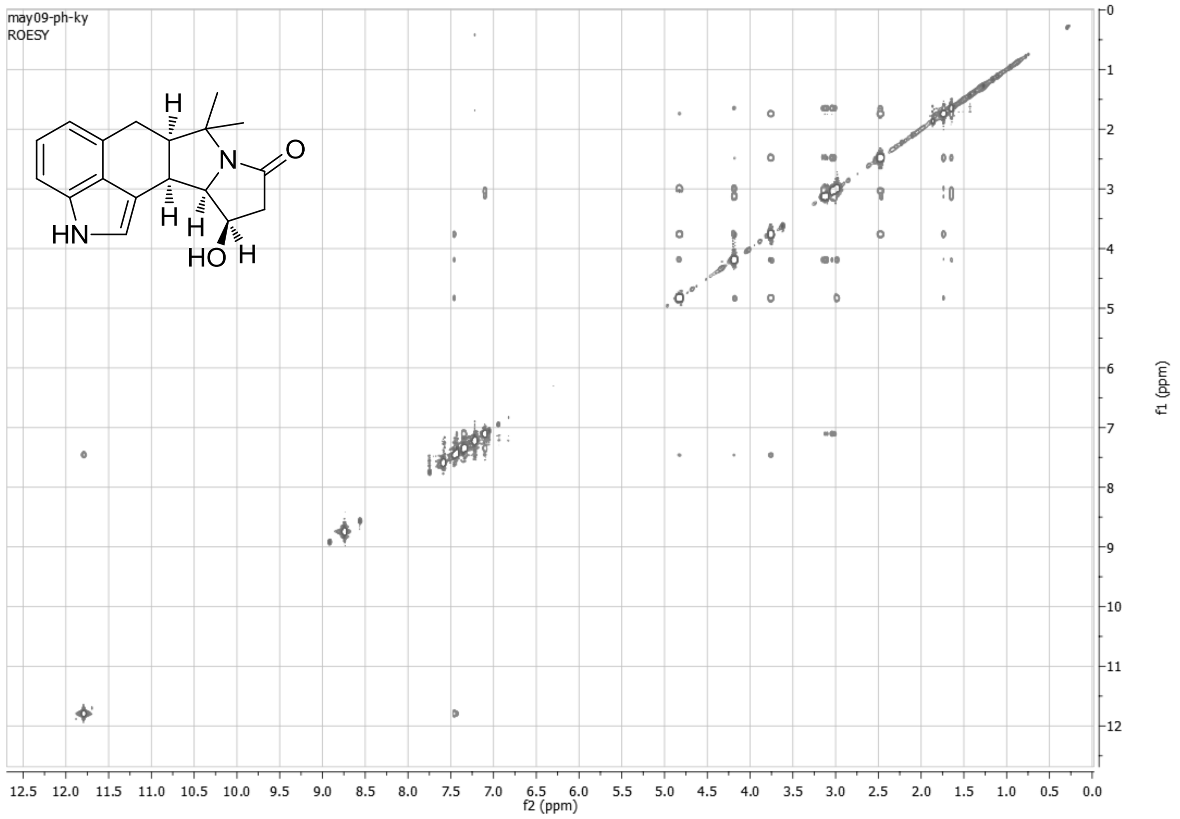

Figure S13. ^{1}H NMR spectrum of Amycolactam (**3**) at 600 MHz in pyridine-d_5.

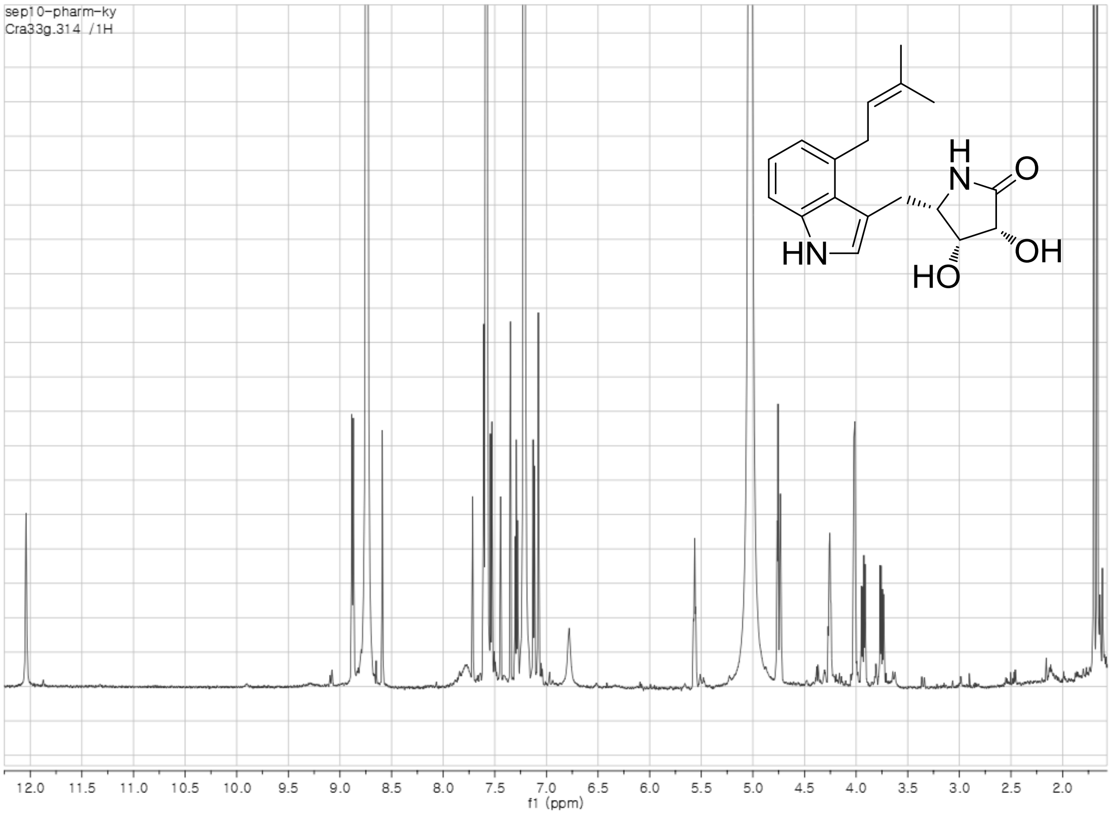

Figure S14. ^{1}H–^{1}H COSY NMR spectrum of Amycolactam (**3**) at 600 MHz in pyridine-d_5.

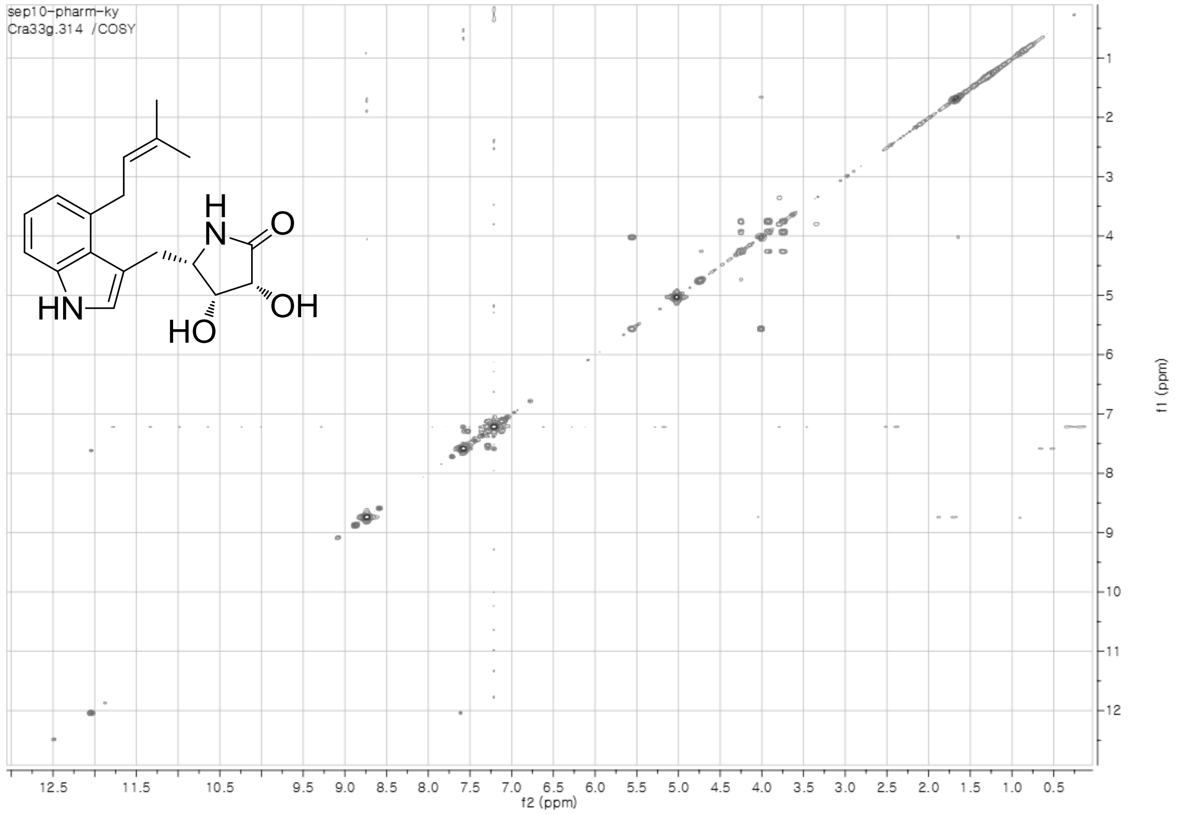

Figure S15. HSQC NMR spectrum of Amycolactam (**3**) at 600 MHz in pyridine-d_5.

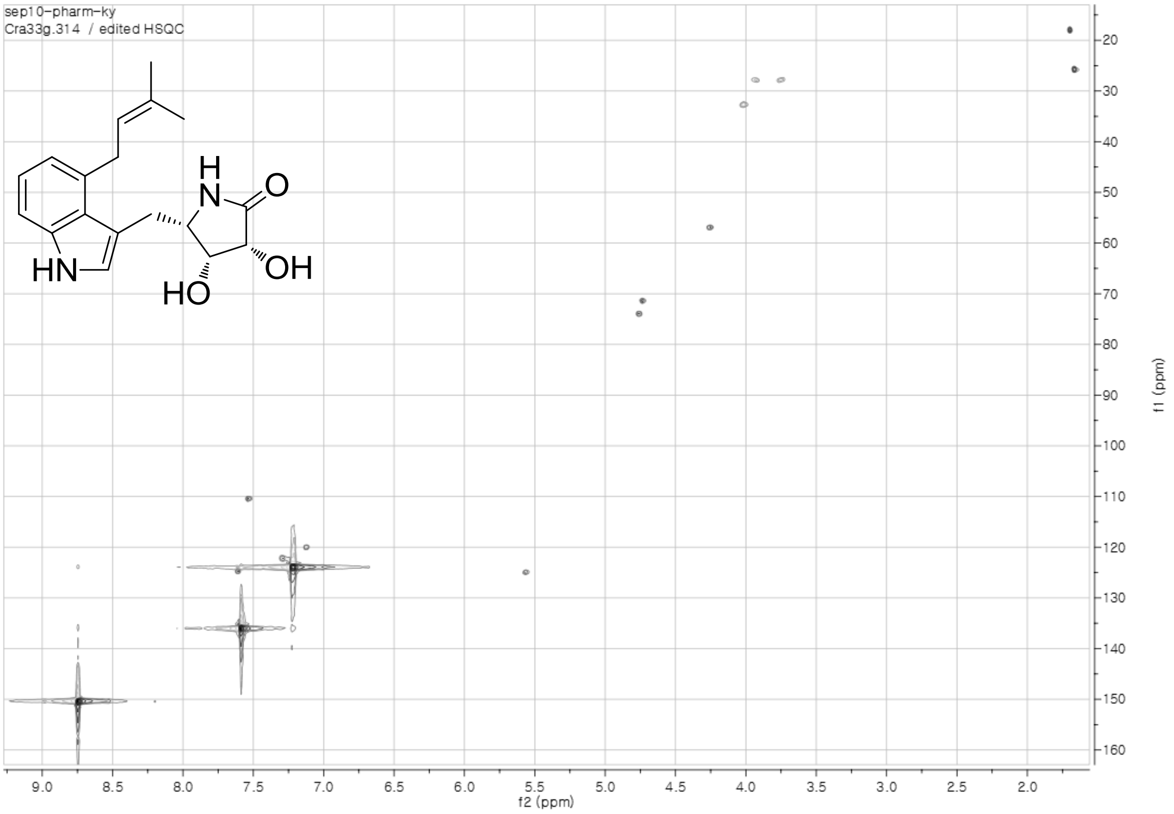

Figure S16. HMBC NMR spectrum of Amycolactam (**3**) at 600 MHz in pyridine-d_5.

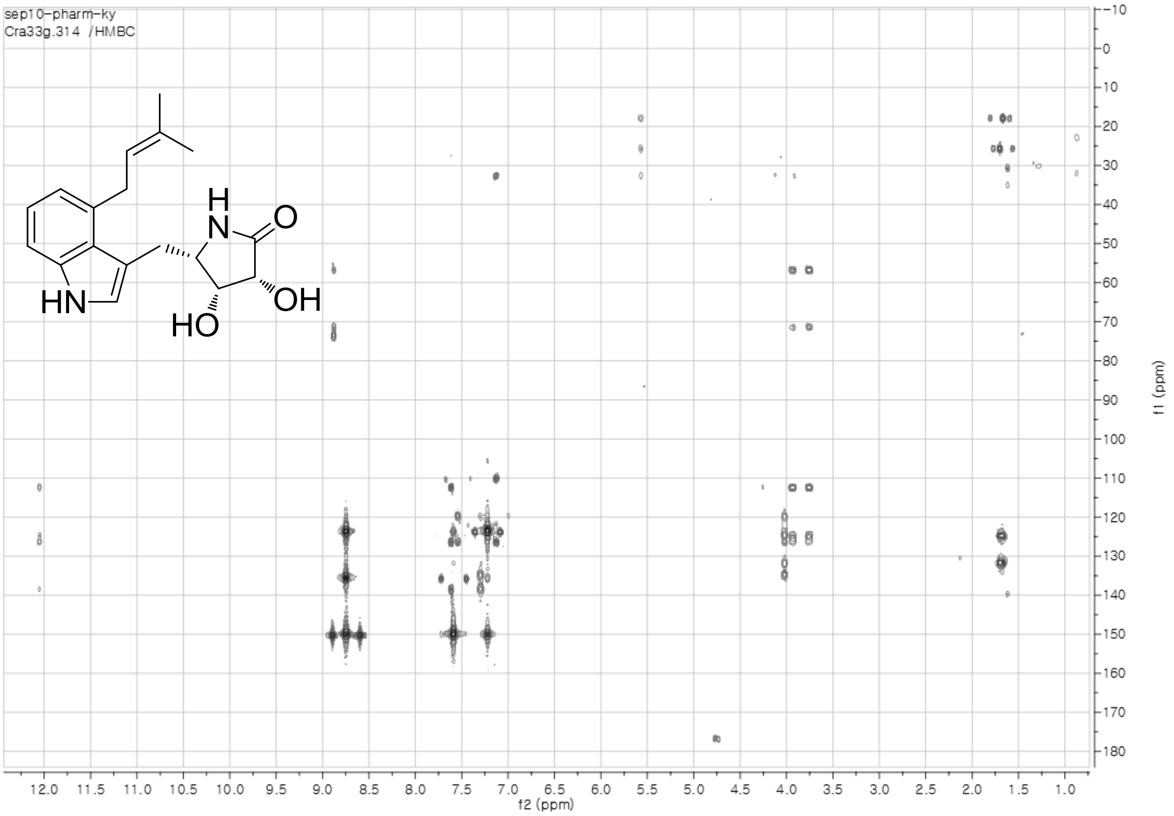

Figure S17. ^{1}H–^{1}H ROESY NMR spectrum of Amycolactam (**3**) at 600 MHz in pyridine-d_5.

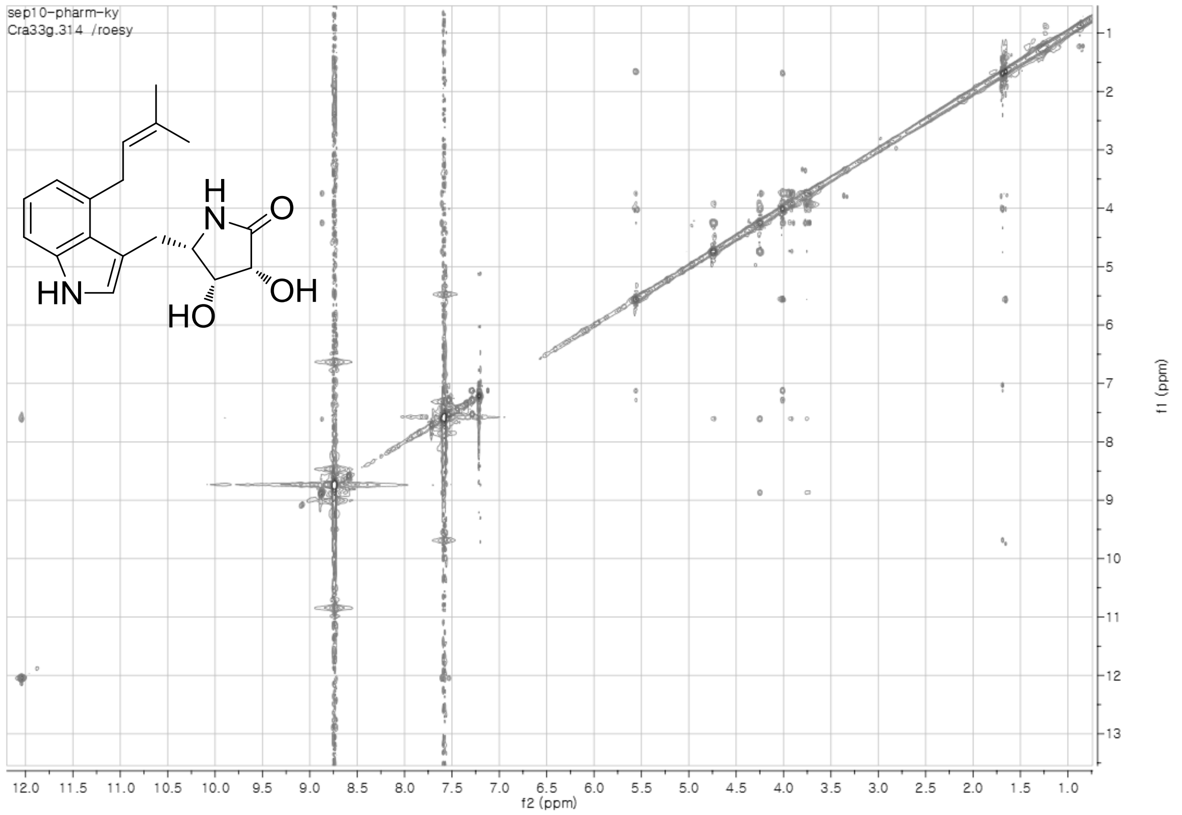

Figure S18. ^{1}H NMR spectrum of *S*-MTPA ester (**4**) for Amycocyclopiazonic acid (**2**) at 600 MHz in pyridine-d_5.

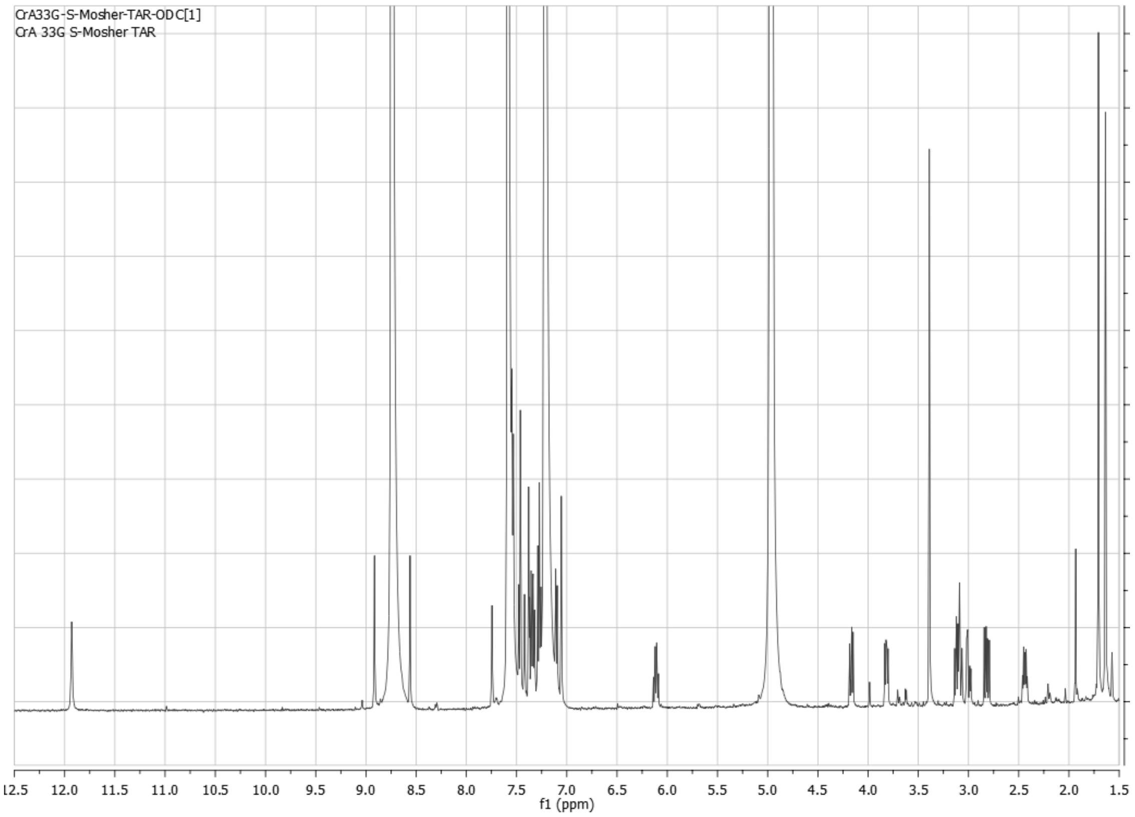

Figure S19. ^{1}H–^{1}H COSY NMR spectrum of *S*-MTPA ester (**4**) for Amycocyclopiazonic acid (**2**) at 600 MHz in pyridine-d_5.

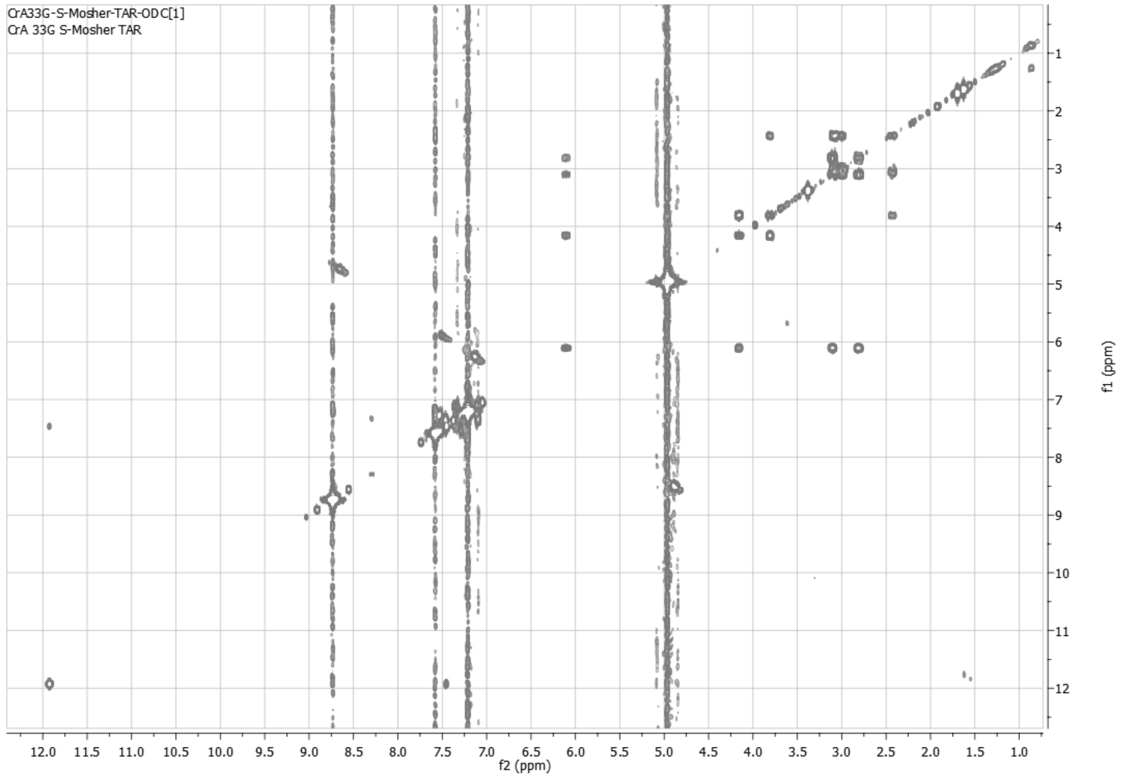

Figure S20. ^{1}H NMR spectrum of *R*-MTPA ester (**5**) for Amycocyclopiazonic acid (**2**) at 600 MHz in pyridine-d_5.

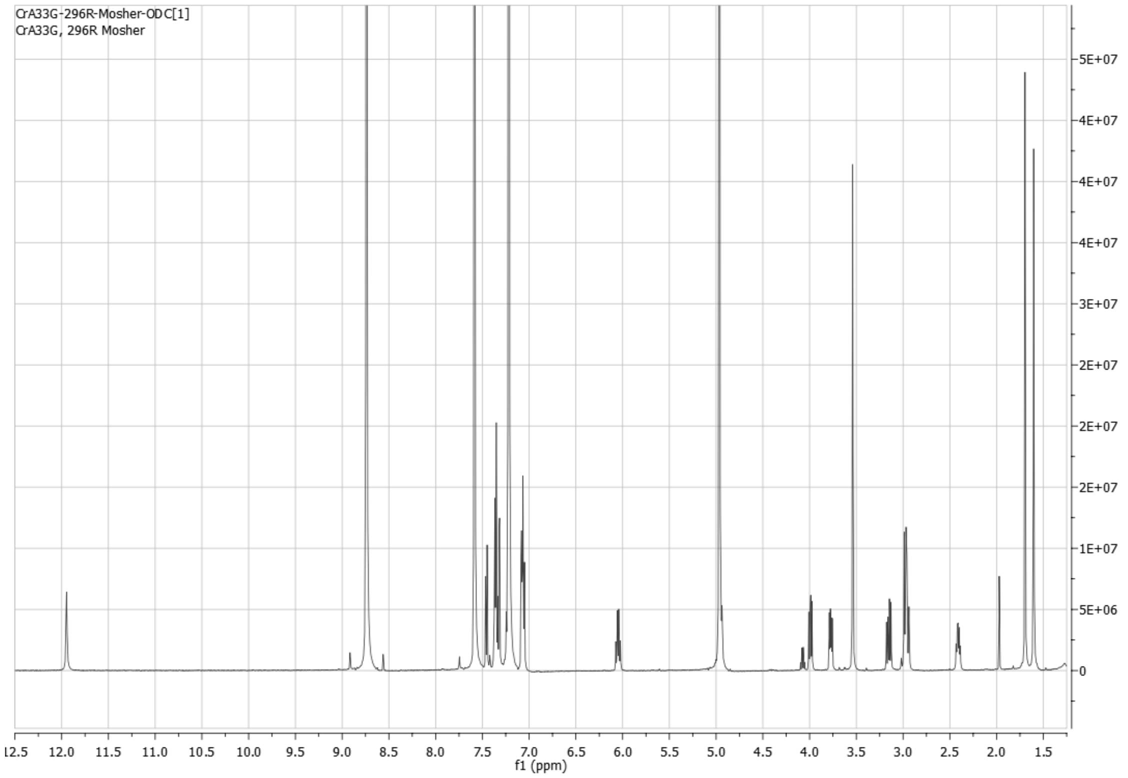

Figure S21. ^{1}H–^{1}H COSY NMR spectrum of *R*-MTPA ester (**5**) for Amycocyclopiazonic acid (**2**) at 600 MHz in pyridine-d_5.

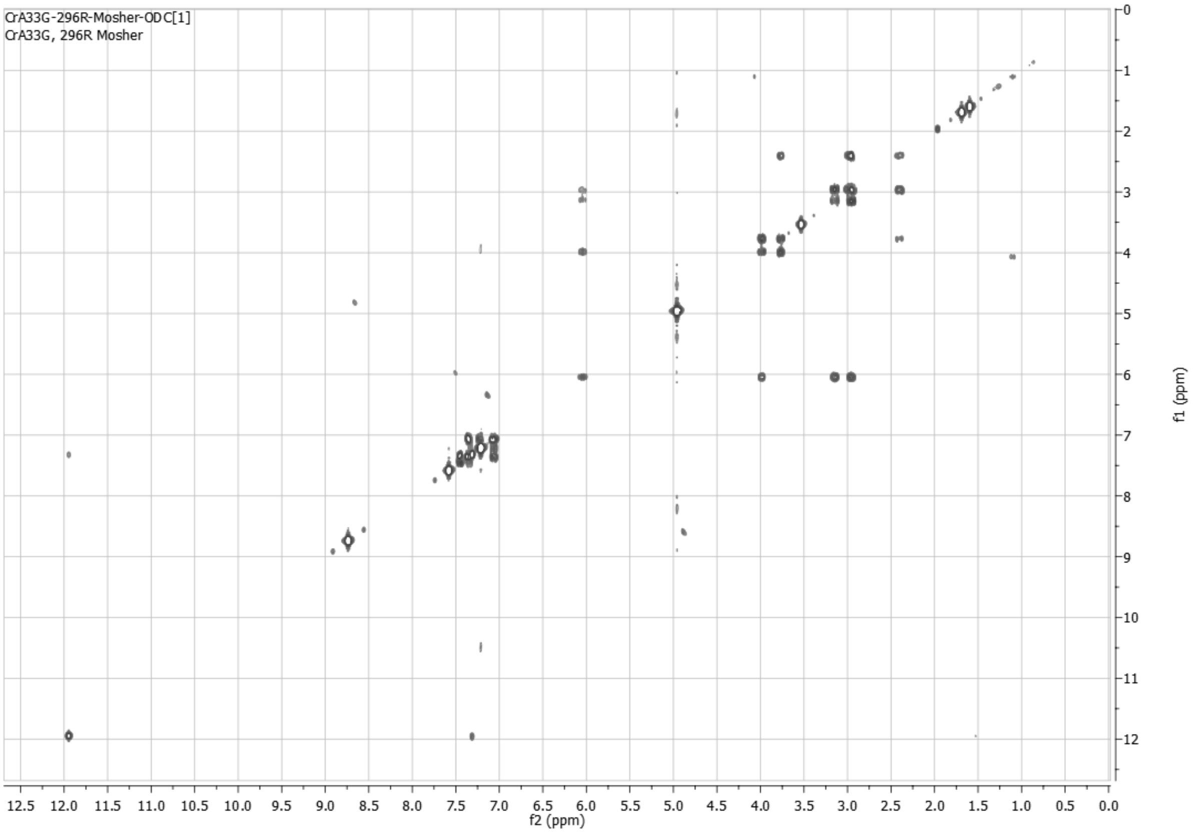

Figure S22. The calculated ECD spectra of C8 isomer of amycofuran (**1**) aglycone.

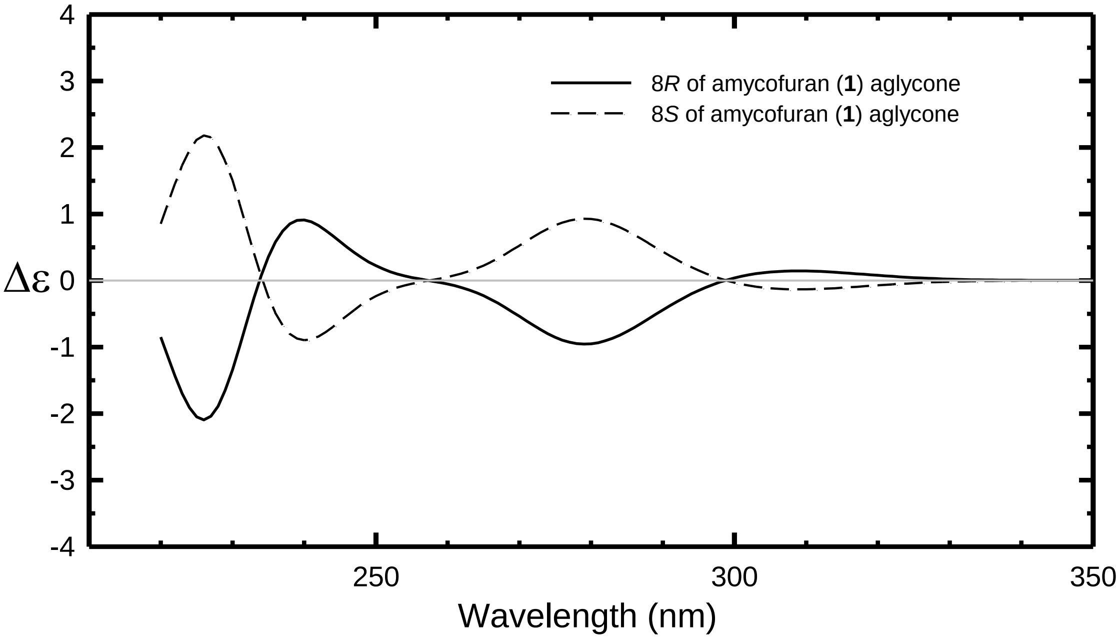

Figure S23. Key ROESY correlations of amycofuran (**1**).

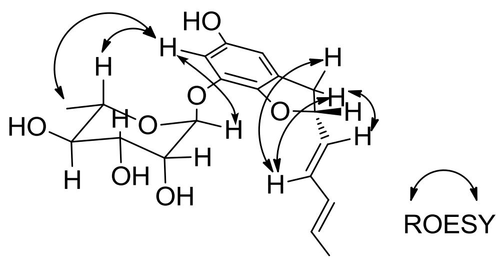

Figure S24. Measured CD and calculated ECD spectra of **3**.

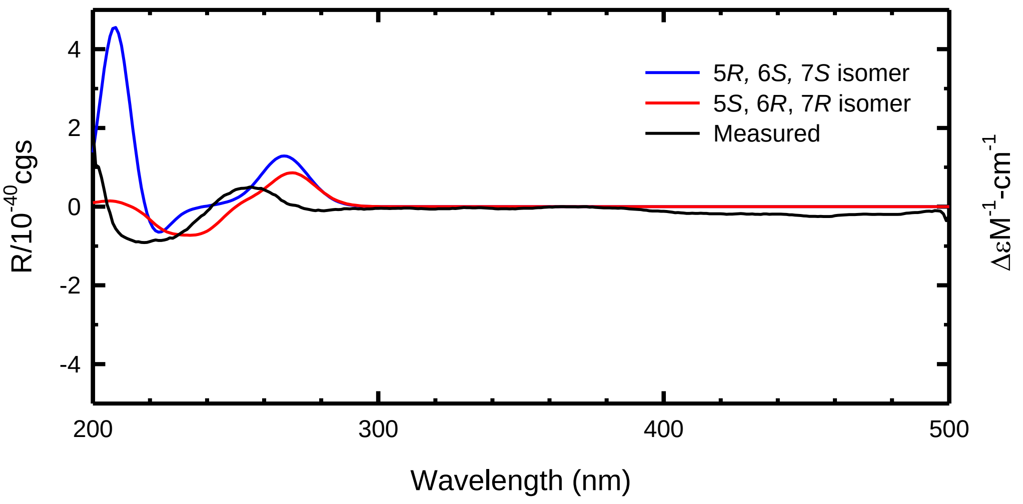

S1. Computational Calculation

S1.1. Amycofuran (1)

Parameters of Level DFT
DFT settings (Functional B3-LYP / Gridsize M3)
Total energy: −1264.76076883502 Ha
Geometry optimization options (Energy 10^{-6} Hartree, Gradient norm $|dE / dxyz| = 10^{-3}$ Hartree/Bohr)

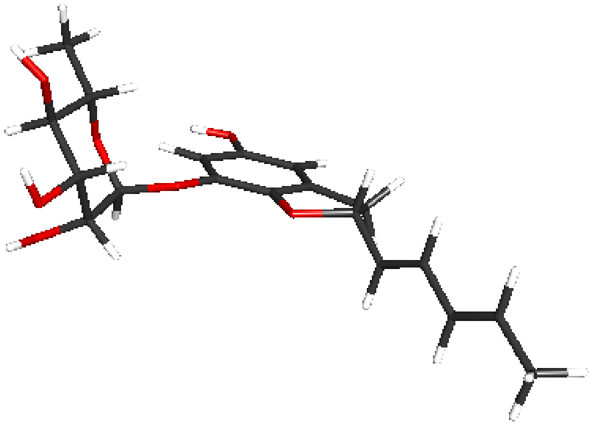

Energy minimized conformation of Amycofuran (**1**) at the B3LYP/def-SV(P).

*S1.2. Amycofuran (**1**) aglycone*

Parameters of Level DFT
DFT settings (Functional B3-LYP / Gridsize M3)
Total energy: −728.43410092823 Ha
Geometry optimization options (Energy 10^{-6} Hartree, Gradient norm $|dE / dxyz| = 10^{-3}$ Hartree/Bohr)

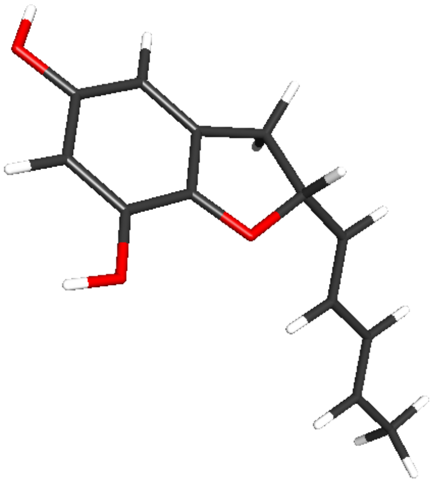

Energy minimized conformation of Amycofuran (**1**) aglycone at the B3LYP/def-SV(P).

*S1.3. Amycolactam (**3**)*

Parameters of Level DFT
DFT settings (Functional B3-LYP / Gridsize M3)
Total energy: −1032.90479525856 Ha
Geometry optimization options (Energy 10^{-6} Hartree, Gradient norm $|dE / dxyz| = 10^{-3}$ Hartree/Bohr)

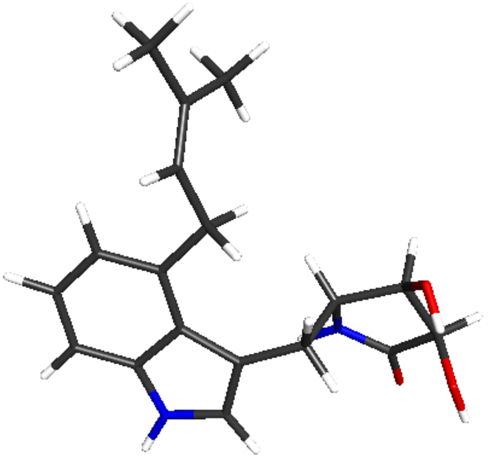

Energy minimized conformation of Amycolactam (**3**) at the B3LYP/def-SV(P).

Table S1. Energy minimized coordinates of Amycofuran (**1**) at the basis set def-SV(P) for all atoms (Å).

Number	Element	X	Y	Z
1	C	0.4048	0.3218	7.2143
2	C	0.3885	−0.5866	8.2881
3	C	1.2242	−1.7155	8.279
4	C	2.0813	−1.9491	7.1995
5	C	2.0886	−1.04	6.1383
6	C	1.2625	0.0794	6.141
7	C	1.5394	0.8485	4.8693
8	C	2.4156	−0.1645	4.0599
9	H	2.0937	1.7824	5.0802
10	H	0.6238	1.1228	4.3181
11	O	2.8764	−1.1468	5.0259
12	C	1.6734	−0.822	2.9244
13	H	3.3252	0.3295	3.6741
14	C	1.5438	−2.149	2.7355
15	H	1.2316	−0.1196	2.2023
16	C	0.8259	−2.765	1.6277
17	H	2.0169	−2.8394	3.4433
18	C	0.7126	−4.1003	1.476
19	H	0.361	−2.0937	0.8902
20	C	−0.0111	−4.7863	0.3563
21	H	1.1856	−4.7446	2.2321
22	H	0.6677	-5.4535	−0.2118
23	H	−0.4517	−4.064	−0.3539
24	H	−0.8281	−5.4299	0.7401
25	H	−0.2489	1.2014	7.2256
26	H	1.2068	−2.4162	9.1156
27	O	−0.4244	−0.4216	9.3722
28	H	−0.9501	0.3881	9.2655
29	O	2.9325	−3.0263	7.1588
30	C	2.4172	−4.2507	6.6984
31	O	2.2061	−5.0758	7.8259
32	C	1.7164	−6.3956	7.5372
33	C	0.2594	−6.4059	7.0693
34	H	−0.3595	−5.8402	7.7878
35	H	−0.1145	−7.446	7.043
36	H	0.1335	−5.9776	6.0638
37	C	2.7379	−7.1071	6.6236
38	H	1.7681	−6.9078	8.5123
39	C	3.0632	−6.2777	5.3541
40	H	2.3332	−8.0871	6.3112
41	O	3.9246	−7.3876	7.347
42	C	3.4693	−4.8613	5.7669
43	O	1.942	−6.199	4.4832

Table S1. *Cont.*

44	H	3.923	−6.7486	4.8408
45	H	4.3937	−4.9521	6.3752
46	O	3.6956	−4.0685	4.633
47	H	1.4713	−4.0755	6.1529
48	H	4.1188	−6.614	7.9065
49	H	1.8197	−7.0615	4.0544
50	H	3.7488	−3.1317	4.9089

Table S2. Energy minimized coordinates of Amycofuran (**1**) aglycone at the basis set def-SV(P) for all atoms (Å).

Number	Element	X	Y	Z
1	C	−3.3092	1.8012	−0.0505
2	C	−4.4347	0.9873	−0.2461
3	N	−4.375	−0.3889	0.0313
4	C	−3.2012	−0.9831	0.5137
5	C	−2.0836	−0.1576	0.7088
6	C	−2.1414	1.2051	0.4335
7	C	−0.7613	1.7681	0.688
8	C	−0.0815	0.5886	1.454
9	C	−0.761	2.6963	1.2842
10	H	−0.2383	1.9799	−0.2644
11	H	−0.8678	−0.583	1.1621
12	H	1.3639	0.3674	1.1256
13	C	−0.1829	0.779	2.5438
14	C	1.873	−0.7413	0.5563
15	H	2.0284	1.2022	1.3894
16	H	3.2827	−0.9297	0.2406
17	C	1.1882	−1.5634	0.3152
18	C	3.7914	−2.0428	−0.3245
19	H	3.9603	−0.0985	0.4858
20	H	5.2351	−2.2661	−0.6627
21	H	3.1007	−2.8652	−0.5629
22	C	5.6402	−3.1519	−0.134
23	H	5.8583	−1.3943	−0.3955
24	H	5.3686	−2.4635	−1.7451
25	H	−3.351	2.8734	−0.271
26	H	−5.2735	−0.9928	−0.1311
27	C	−5.6255	1.4737	−0.7061
28	C	−5.5448	2.429	−0.8624
29	C	−3.0929	−2.3074	0.7963
30	C	−3.9407	−2.7455	0.612

Table S3. Energy minimized coordinates of Amycolactam (**3**) at the basis set def-SV(P) for all atoms (Å).

Number	Element	X	Y	Z
1	C	6.4428	1.1437	5.7331
2	C	7.6772	0.9278	5.1547
3	N	8.323	−0.1157	5.7787
4	C	7.522	−0.6082	6.7848
5	C	6.3181	0.1613	6.8007
6	C	5.3291	−0.145	7.7799
7	C	5.585	−1.2068	8.6484
8	C	6.7798	−1.9586	8.6005
9	C	7.77	−1.6657	7.6728
10	H	4.8314	−1.4656	9.3966
11	H	6.9256	−2.7787	9.3109
12	H	8.7043	−2.2343	7.6324
13	C	4.0372	0.6619	7.8849
14	C	3.2131	0.4175	9.1226
15	H	4.3041	1.7354	7.8699
16	H	3.4318	0.5003	6.9756
17	C	1.9708	−0.0939	9.2167
18	C	1.2867	−0.2222	10.5587
19	H	1.0135	−1.2758	10.7684
20	H	0.341	0.3552	10.5797
21	H	1.9241	0.137	11.3849
22	C	1.144	−0.5769	8.049
23	H	0.2036	0.0035	7.9681
24	H	0.8461	−1.6338	8.1955
25	H	1.6667	−0.5085	7.0819
26	H	3.7017	0.7195	10.06
27	C	5.4645	2.1723	5.2264
28	C	4.4157	1.6026	4.2441
29	C	3.4619	2.6914	3.6431
30	C	4.1191	3.0733	2.304
31	C	4.9885	1.8589	1.9427
32	N	5.0096	1.0425	3.0349
33	H	3.8265	0.8256	4.7606
34	O	5.5878	1.7094	0.8949
35	O	4.9573	4.1999	2.5365
36	H	5.5168	4.3336	1.7509
37	H	3.3872	3.2898	1.5044
38	O	3.2067	3.7863	4.4753
39	H	3.8978	4.451	4.2923
40	H	2.4925	2.2087	3.4313
41	H	5.641	0.2458	3.0673
42	H	9.2492	−0.4536	5.552
43	H	4.9171	2.6599	6.0475
44	H	6.0186	2.9692	4.7002
45	H	8.1509	1.4647	4.332

Figure S25. Phylogenetic tree based on 16S rDNA sequences of *Amycolatopsis* sp. Cra33g.

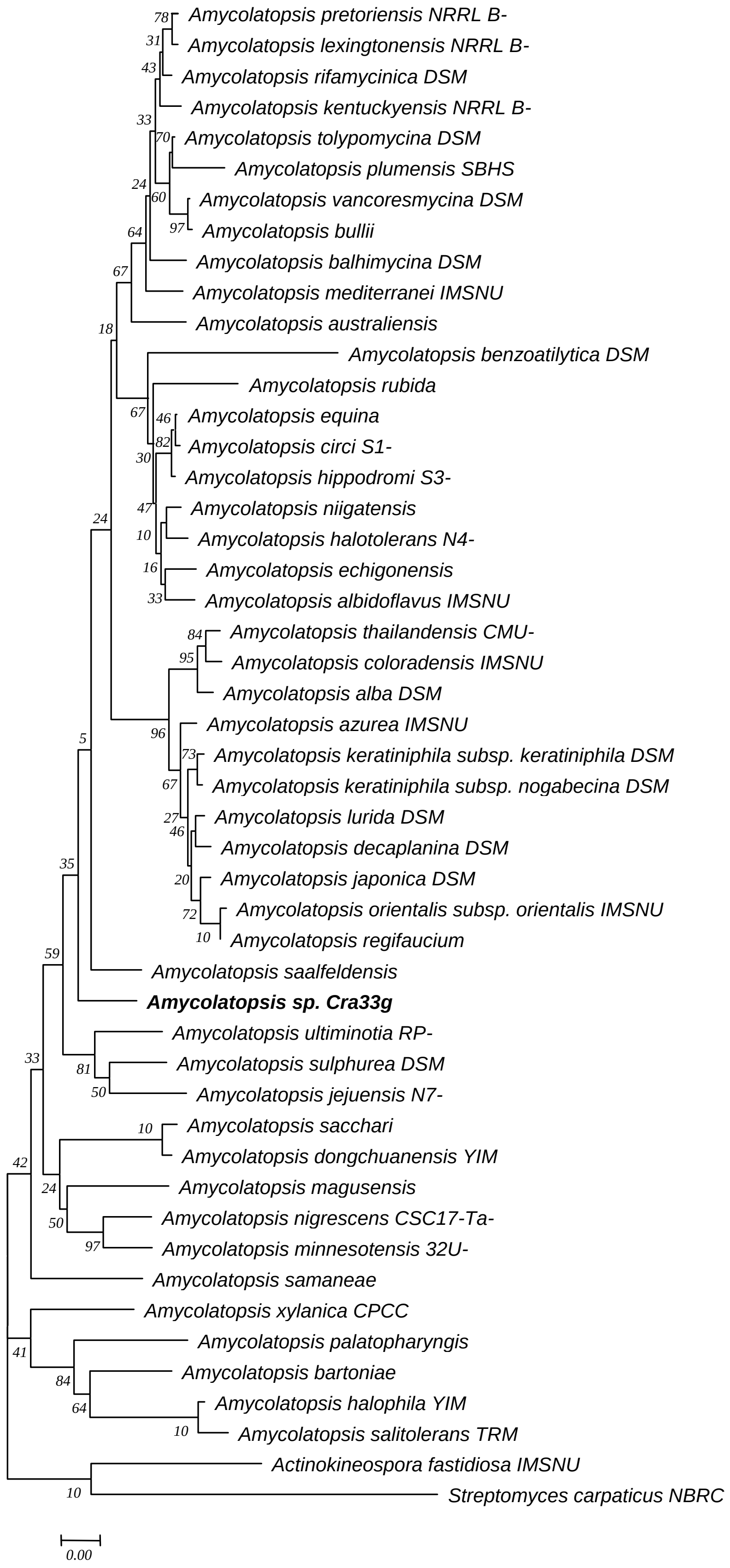

Figure S26. 16S rDNA sequence data of *Amycolatopsis* sp. Cra33g.

TCAGGACGAACGCTGGCGGCGTGCTTAACACATGCAAGTCGAACGCTGAACCGGTTTCGG
CCGGGGATGAGTGGCGAACGGGTGAGTAACACGTGGGTAATCTGCCCTGTACTCTGGGATA
AGCCTGGGAAACTGGGTCTAATACCGGATATGACCATTACAGGCATCTGTGGTGGTGGAAA
GTTCCGGCGGTATGGGATGAACCCGCGGCCTATCAGCTTGTTGGTGGGGTAATGGCCTACC
AAGGCGACGACGGGTAGCCGGCCTGAGAGGGTGACCGGCCACACTGGGACTGAGACACG
GCCCAGACTCCTACGGGAGGCAGCAGTGGGGAATATTGCACAATGGGCGCAAGCCTGATG
CAGCGACGCCGCGTGAGGGATGACGGCCTTCGGGTTGTAAACCTCTTTCGCCAGGGACGA
AGCGCAAGTGACGGTACCTGGATAAGAAGCACCGGCTAACTACGTGCCAGCAGCCGCGGT
AATACGTAGGGTGCGAGCGTTGTCCGGAATTATTGGGCGTAAAGAGCTCGTAGGCGGTTTG
TCGCGTCGGCTGTGAAATCTGGAGGCTTAACCTTCAGCGTGCAGTCGATACGGGCAGACTT
GAGTTCGGCAGGGGAGACTGGAATTCCTGGTGTAGCGGTGAAATGCGCAGATATCAGGAG
GAACACCGGTGGCGAAGGCGGGTCTCTGGGCCGATACTGACGCTGAGGAGCGAAAGCGT
GGGGAGCGAACAGGATTAGATACCCTGGTAGTCCACGCTGTAAACGTTGGGCGCTAGGTGT
GGGCGACATTCCACGTTGTCCGTGCCGTAGCTAACGCATTAAGCGCCCCGCCTGGGGAGTA
CGGCCGCAAGGCTAAAACTCAAAGGAATTGACGGGGGCCCGCACAAGCGGCGGAGCATG
TGGATTAATTCGATGCAACGCGAAGAACCTTACCTGGGCTTGACATGCGCCAGACATCCCC
AGAGATGGGGCTTCCCTTGTGGTTGGTGTACAGGTGGTGCATGGCTGTCGTCAGCTCGTGT
CGTGAGATGTTGGGTTAAGTCCCGCAACGAGCGCAACCCTTATCCTACGTTGCCAGCGCGT
CATGGCGGGGACTCGTGGGAGACTGCCGGGGTCAACTCGGAGGAAGGTGGGGATGACGT
CAAGTCATCATGCCCCTTATGTCCAGGGCTTCACACATGCTACAATGGCTGGTACAGAGGG
CTGCGATACCGCGAGGTGGAGCGAATCCCTTAAAGCCGGTCTCAGTTCGGATCGCAGTCTG
CAACTCGACTGCGTGAAGTCGGAGTCGCTAGTAATCGCAGATCAGCAACGCTGCGGTGAAT
ACGTTCCCGGGCCTTGTACACACCGCCCGTCACGTCATGAAAGTCGGTAACACCCGAAGC
CCATGGCCCAACCCGCAAGGGAGGGAGTGGTCGAAGGTGGGACTGGCGATTGG

© 2014 by the authors; licensee MDPI, Basel, Switzerland. This article is an open access article distributed under the terms and conditions of the Creative Commons Attribution license (http://creativecommons.org/licenses/by/3.0/).
